# Cardiovascular health promotion: A systematic review involving effectiveness of faith-based institutions in facilitating maintenance of normal blood pressure

**DOI:** 10.1371/journal.pgph.0001496

**Published:** 2023-01-20

**Authors:** Abayomi Sanusi, Helen Elsey, Su Golder, Osayuwamen Sanusi, Adejoke Oluyase

**Affiliations:** 1 Department of Health Sciences, University of York, York, United Kingdom; 2 National Health Service, York, United Kingdom; 3 Cicely Saunders Institute of Palliative Care & Rehabilitation, King’s College London, London, United Kingdom; Babcock University, NIGERIA

## Abstract

Globally, faith institutions have a range of beneficial social utility, but a lack of understanding remains regarding their role in cardiovascular health promotion, particularly for hypertension. Our objective was assessment of modalities, mechanisms and effectiveness of hypertension health promotion and education delivered through faith institutions. A result-based convergent mixed methods review was conducted with 24 databases including MEDLINE, Embase and grey literature sources searched on 30 March 2021, results independently screened by three researchers, and data extracted based on behaviour change theories. Quality assessment tools were selected by study design, from Cochrane risk of bias, ROBINS I and E, and The Joanna Briggs Institute’s Qualitative Assessment and Review Instrument tools. Twenty-four publications contributed data. Faith institution roles include cardiovascular health/disease teaching with direct lifestyle linking, and teaching/ encouragement of personal psychological control. Also included were facilitation of: exercise/physical activity as part of normal lifestyle, nutrition change for cardiovascular health, cardiovascular health measurements, and opportunistic blood pressure checks. These demand relationships of trust with local leadership, contextualisation to local sociocultural realities, volitional participation but prior consent by faith / community leaders. Limited evidence for effectiveness: significant mean SBP reduction of 2.98 mmHg (95%CI -4.39 to -1.57), non-significant mean DBP increase of 0.14 mmHg (95%CI -2.74 to +3.01) three months after interventions; and significant mean SBP reduction of 0.65 mmHg (95%CI -0.91 to -0.39), non-significant mean DBP reduction of 0.53 mmHg (95%CI -1.86 to 0.80) twelve months after interventions. Body weight, waist circumference and multiple outcomes beneficially reduced for cardiovascular health: significant mean weight reduction 0.83kg (95% CI -1.19 to -0.46), and non-significant mean waist circumference reduction 1.48cm (95% CI -3.96 to +1.00). In addressing the global hypertension epidemic the cardiovascular health promotion roles of faith institutions probably hold unrealised potential. Deliberate cultural awareness, intervention contextualisation, immersive involvement of faith leaders and alignment with religious practice characterise their deployment as healthcare assets.

## Introduction

Hypertension, the systolic/diastolic blood pressure of 140/90 mmHg or higher [1–3], is the most important risk factor for cardiovascular morbidity and mortality; and a progressively worsening epidemic in Sub Saharan Africa (SSA) [4–10]. Large hypertension outcome inequalities exist between countries with organised, well-functioning healthcare and public health systems, and populations of SSA with less well organised and funded public health systems [11].

Faith institutions are non-profit entities characterised by expressions of religious creed and implementation of religious worship [12, 13]. They comprise social and cultural networks based on religious traditions or worship practices, and may be involved in providing social services for people within their networks or the wider society. Faith institutions addressed in this review are limited to religious organisations operating worship assembly and social services, not including faith-based higher education institutions and healthcare facilities.

Globally faith institutions are culturally and socio-politically influential [14–17], occupying a peculiar position of influence that potentially frames them as healthcare assets [18–23]. They are part of the structure of societies, and are seen as socially acceptable to contribute to health education and risk reduction campaigns [24–27].

From research and housing to healthcare, there is evidence of involvement and influence of faith-based institutions in a wide range of socially beneficial programmes [19, 28–38]. Knowledge gaps however remain on their role in cardiovascular risk reduction. Specifically, how faith institutions have contributed to hypertension health promotion and facilitation of hypertension screening. Similarly, evidence is needed to understand the features likely to make faith-institution based hypertension interventions acceptable and contextually optimal- particularly for low resource settings and settings without well organised healthcare and public health systems.

The following questions are therefore addressed: What is the evidence for the role of faith-institutions in cardiovascular health promotion to reduce hypertension or maintain normal blood pressure? What are the characteristics of those roles?

The objectives are to:

- Summarise the evidence that address faith institution facilitated hypertension risk reducing activities toward achievement or maintenance of normal blood pressure in adults.
- Enumerate and describe the roles played by faith institutions in influencing hypertension risk reducing behaviour of their adult participants through hypertension health promotion, education, blood pressure measurements and other functions influencing hypertension risk reduction.
- Identify and describe the key common characteristics of these interventions including any hypertension health promotion, education and blood pressure measuring procedures.
- Assess the effectiveness of the interventions delivered by faith institutions to achieve or maintain normal blood pressure.

## Methods

The review was registered on the National Institute for Health Research PROSPERO database (CRD42021228938). In accordance with the recommended systematic review process, the protocol was followed and no changes were made. For quantitative synthesis, random effects model meta-analyses were performed using all available primary and secondary outcomes data from the randomised controlled trials. All the randomised controlled studies that had the required data were included in the meta-analyses regardless of risk of bias assessment. Given the relative dearth of studies coupled with clinical and methodological heterogeneity between available ones, attempts such as network meta-analysis or combination of data from randomised studies, nonrandomised studies, before-and-after studies, and cohort studies, were not made. For qualitative synthesis the Joanna Briggs Institute’s integrative meta-aggregation approach was utilised, framing findings on the Diffusion of Innovation theory and the Communication–Behaviour change model. To complete the mixed methods approach using the results based convergent design, a final synthesis was performed of the quantitative and qualitative findings [39].

### Eligibility

All literature addressing hypertension risk reduction targeting adults within faith institutions were eligible. These included literature on hypertension health promotion, hypertension information or education, hypertension risk modification involving weight reduction, increasing physical activity, adoption of healthier diets, hypertension monitoring and engagement with healthcare system. Studies reporting prevention, monitoring, interventions and other activities that are based exclusively on faith, faith-related practices, rituals, activities inexplicable by or incompatible with medical science, or controversial practices were excluded. Studies focussing on yoga were excluded because yoga is also sometimes regarded a physical and spiritual practice, rather a means of providing health information or education for behaviour change [40–43].

Study designs eligible include randomised controlled and non-controlled studies, nonrandomised studies including before and after studies, observational studies, qualitative studies, surveys and reports. The inclusion and exclusion criteria of studies are outlined in Table 1.

**Table 1:** Outline of criteria for the inclusion and exclusion of studies.

	Eligible studies for inclusion	Ineligible studies
P (Population)	• Studies carried out within or in collaboration with a faith-based institution or groups of faith-based institutions • Studies including interventions aimed at adult participants (with adults described as individuals aged at least 16 or 18, depending on the setting)	• Studies exclusively reporting interventions on and data from children or non-adult participants.
I (intervention)	• Studies reporting on at least one hypertension prevention, monitoring or treatment activity • Studies including interventions or hypertension related activities that are strictly conventional, universal and based on modern health/medical science	• Studies not reporting on any hypertension intervention or hypertension risk factor intervention • Studies reporting on ritual, spiritual, unscientific or unverifiable activities, or activities with no evidence base in blood pressure reduction. • Studies reporting prevention, monitoring, interventions and other activities that are based exclusively on faith, faith-related practices, rituals, activities inexplicable by or incompatible with health/medical science, or controversial practices (e.g. Yoga, Meditation practices etc.). • Studies incorporating unclassifiable, unstated or unclear interventions.
C (Comparison)	There are no comparators.	
O (Outcome)	Primary: • Reduction of adult blood pressure measurements from hypertensive to normal blood pressure levels, or maintenance of normal blood pressure. Secondary: • Hypertension risk modifying outcomes including weight reduction, increased physical activity, adoption of healthier diets, increased hypertension awareness, increased hypertension monitoring, increased engagement with healthcare system. • Process indicators of the interventions including intervention acceptability, intervention uptake and participant satisfaction.	

### Search methods

The following information sources were searched in March 2021, and the results indicating the available literature from their inception to end of March 2021: The Cochrane Library, Epistemonikos, Campbell Library, The International Initiative for Impact Evaluation (3IE), Database of Promoting Health Effectiveness Reviews (DoPHER), Database of Abstracts of Reviews of Effects (DARE), and the Health Technology Assessment (HTA) Database.

Others were MEDLINE, EMBASE, PsycINFO, Cumulative Index to Nursing and Allied Health Literature (CINAHL), Allied and Complementary Medicine Database (AMED), Oxford Bibliographies on Public Health, Scopus, Trials Register of Promoting Health Interventions (TRoPHI), Applied Social Sciences Index & Abstracts (ASSIA), and BiblioMap—The EPPI-Centre database of health promotion research. For grey literature the following databases were searched: The Bielefield Academic Search Engine- BASE, The King’s Fund Library Database, OpenGrey multidisciplinary European database, Open Directory of Open Access Repositories–OpenDOAR, and the National Institute for Health Research—NIHR website and the British Library Catalogue. The British Library E-theses online service database (EThOS) and the ProQuest Dissertations and Theses database were searched. Reference checking was performed on all publications screened eligible for inclusion.

The search strategy combined descriptors for the study population, intervention of interest and the settings are outlined in Tables 2 and 3. The retrieved search results were imported into EndNote and de-duplicated.

**Table 2:** OVID medline search strategy–inception till 31^st^ March 2021.

1	Faith or religio* or christian* or islam* or Jew* or Judaism or Hindu* or buddh* or theis*.mp
2	Institution* or organisation* or associatio* or organizatio* or church or mosque* or synagogue or temple or tabernacle or assembly or congregation*.mp
3	organizations/
4	faith-based organizations/
5	exp hypertension/
6	Hypertension.mp
7	high blood pressure.mp
8	raised blood pressure.mp
9	elevated blood pressure.mp
10	Education or information or promotion or campaign or publicity.mp
11	Measure* or screen* or surveillance or monitor* or evaluat*.mp
12	exp Health Education/
13	Mass Screening/
14	exp Blood Pressure Determination/
15	Blood Pressure/
16	1 and (2 or 3)
17	4 or 16
18	or/5-9
19	or/10-15
20	17 and 18 and 19

**Table 3:** Search terms.

Population (Adult participants of faith-based institutions)	Faith, Religious, Religion(s), Christian, Islam, Judaism, Hinduism, Buddhism, Theisms Institution, Organisation, Association, Network, Congregation
Intervention (Hypertension preventative activities)	Hypertension, High blood pressure, Systemic arterial blood pressure, Essential hypertension Education, Information, Promotion, Campaign, Publicity Measure, Measures, Measurement(s), Screen, Surveillance, Monitor, Monitoring, Evaluate, Evaluation
Comparison	None
Outcome	Normal blood pressure, Normotensive, Controlled blood pressure
Study	RCT, Non-RCT, Observational studies, Qualitative studies, Surveys and Reports

### Screening

Title and abstracts were triple screened independently by three reviewers (AS, AO, OS) using Rayyan [44]. Uncertainties and disagreements were resolved by discussion. A fourth reviewer (SG) was available where disagreements could not be resolved. Following screening, two (AS, OS) independently reviewed the full text of the selected publications.

### Data extraction

Using a Microsoft word form (independently made by AS and then checked by OS), data extracted included: author’s name, publication year, country of intervention, type of faith institution, funding (state/private sector/non-governmental/faith institution), physical location of interventions (limited to faith institution/extends outside faith-institution), intervention scope (hypertension focussed/ broad cardiovascular disease/ broad chronic disease), intention (one-off research, cyclical/periodical, permanent programme), basis (voluntary/ subtle coercion), mechanism of action of the intervention, intervention target level (individual, community or population), agents delivering interventions, frequency of interventions, materials used in intervention delivery, religious component of interventions, actual intervention or combination of interventions (publicity/information campaign, patient education, measurement, monitoring, advice, healthcare system interfacing/referral, risk factor management/non pharmaceutical treatment, pharmaceutical treatment), and data on unforeseen implementation challenges.

### Risk of bias assessment

AS independently conducted the risk of bias assessments and OS checked the assessments. The Cochrane Risk of Bias tool was used for randomised studies [45], ROBINS I (Risk Of Bias In Non-randomised Studies—Interventions) tool for non-randomised studies [46] and The ROBINS–E tool (Risk Of Bias In Non-randomised Studies—Exposures) [47] for observational studies. The Joanna Briggs Institute’s Qualitative Assessment and Review Instrument (QARI) was used to assess the risk of bias of qualitative studies [48, 49] and the Mixed Methods Appraisal tool for mixed methods studies [50].

### Data synthesis

Syntheses were conducted by a single reviewer (AS), and crosschecked by a second (OS). The characteristics of included studies, key characteristics of interventions, and categories of the identified roles of faith institutions are presented. The cardiovascular health promotion and hypertension screening activities described in the included studies as faith institution facilitated roles were categorised.

To synthesise data on characteristics of the roles, the integrative meta-aggregation approach by the Joanna Briggs Institute was undertaken manually using Microsoft word [49]. From the included studies, findings on predefined elements based on the Diffusion of Innovation theory, the Communication–Behaviour change model, and the Template for Intervention Description and Replication (TIDieR) Checklist were collated and combined. From the combined findings, categories were generated on the basis of similarity and meaning of their content. Finally, the categories were combined to generate synthesis statements that integrate the evidence contained in or expressed by the categories.

Evidence of effectiveness of faith institution roles was presented as summary estimates of individual outcome measures where possible. Meta analysis was completed using version 5.4 of Cochrane software Review Manager [51].

Using tables, summary non-aggregated overview of direction of blood pressure change is presented to show indications of the general direction of change of the systolic and diastolic blood pressures. For the multiple distinct secondary outcomes that have an impact on hypertension and cardiovascular health but could not be summarised or aggregated, a tabular representation is presented of their impact.

The final synthesis is presented in tabular form.

## Results

**Fig 1:**
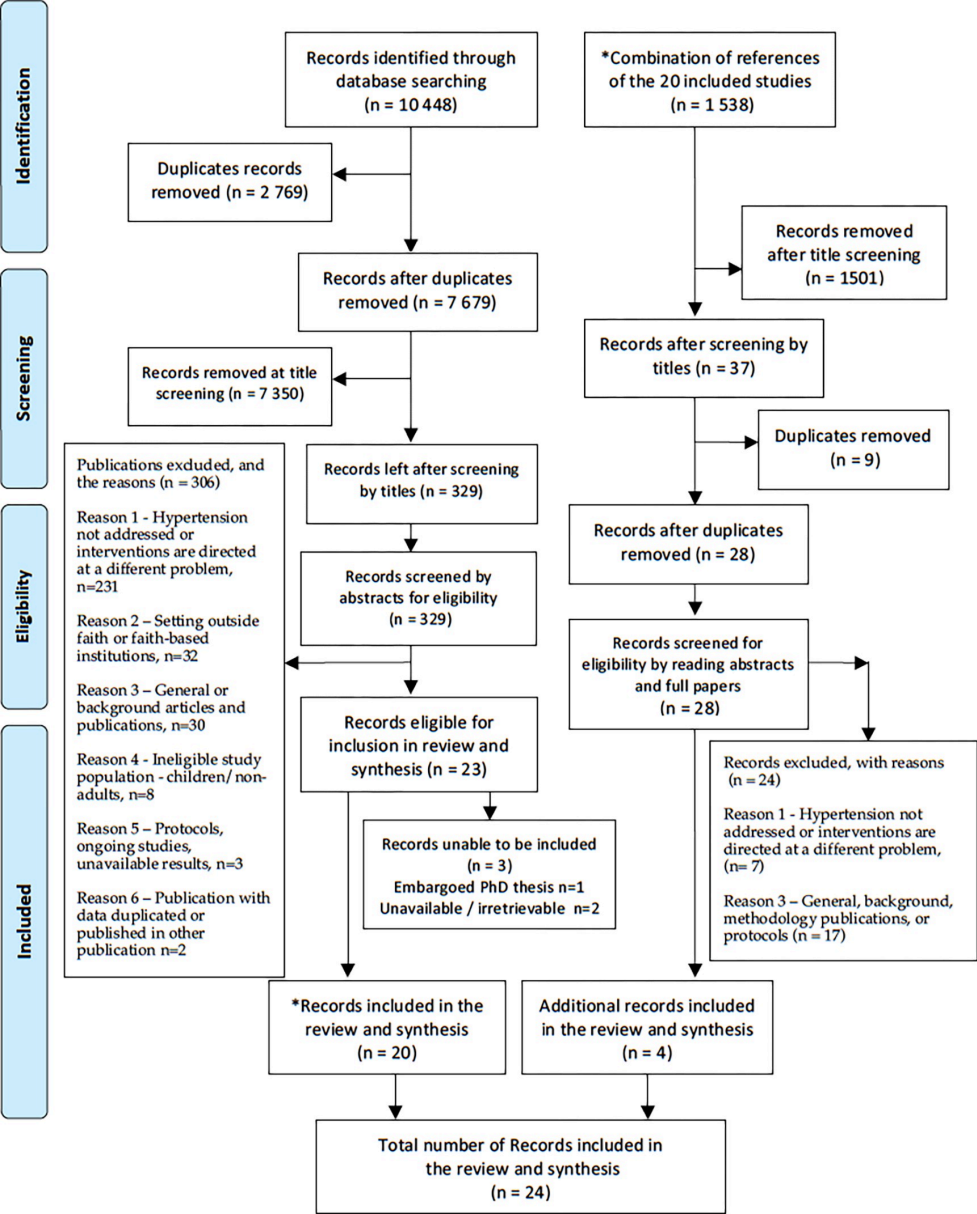
Preferred reporting items for systematic review and meta-analysis flow diagram showing the process of study selection.

Searches retrieved 10 448 records which after de-duplication reduced to 7 679 (Fig 1). After screening by titles 329 records remained and 23 remained after screening by abstract. Out of these, three were unavailable: a PhD thesis under embargo [52], and two abstract publications [53, 54]. This left 20 publications available for inclusion in the review. Reference checking the included studies within these 20 publications retrieved an additional four records, bringing the total number of included publications to twenty-four.

### Characteristics of the included studies

Each study incorporated data from single countries, except one [55] with data from multiple countries. Twenty were set in the United States of America and one study each was set in China, Norway and South Africa [56–58].

Of the 24 studies, 16 were non-randomised, six were randomised controlled studies and two were qualitative studies. All the randomised controlled studies were conducted in the USA. Of the nonrandomised studies, eight were before and after intervention studies, five were uncontrolled longitudinal studies, two were cohort in design and one was a non-randomised controlled study (Table 4).

**Table 4:** Included studies, intervention activities, faith institution roles covered by intervention activities, component mechanisms of the interventions, evidence of effectiveness, and quality ratings of the studies.

Included Studies	Details of interventions		Roles covered by intervention activities		Component mechanisms of the intervention	Evidence of effectiveness	Quality Rating
Author, Year Country (WHO region) Study design	Name of intervention Setting Funding Scope Intention Number of participants	Summary description of the intervention / programme in the study	Promotion & Education	Screening			
**Tucker et al., 2019 USA (WHO Americas region) Randomised Controlled Trial**	Health-Smart AME intervention Church/ Church premises Non Profit Organisation Hypertension risk and chronic diseases Improve health literacy; Increase health promoting behaviour; Reduce weight; Reduce Blood Pressure 172 intervention, 149 control	Church leaders and members, supervised by healthcare professionals, delivered individual and interpersonal level health coaching and physical activity to urban Black / African American adults across all income levels.	✓		Combination of Exercise / Physical Activity with Healthy Lifestyle Coaching, Counselling & Motivation Training	**Systolic Blood Pressure (SBP) decrease** (2.91mmHg, F value = 2.48, p = 0.117–v–Control: reduction of 1.83 mmHg, F = 1.06, p = 0.303). **Diastolic Blood Pressure (DBP) decrease** (0.3mmHg, F value = 0.06, p = 0.815 –v–Control: reduction 2.17 mmHg, F = 3.18, p = 0.076). **Body Weight decrease** (1.69 Lbs., F = 2.95, p = 0.087 –v–Control: reduction of 0.97 Lbs., F value = 1.07, p = 0.303). **Nutrition Label Literacy increase** (1.2 units, F value = 30.89, p<0.001 –v–Control group non-significant decrease of 0.06 units, F value = 0.09, p = 0.76) **Healthy Eating score increase** (0.28, F = 26.32, p<0.001 –v–control group score of 0.08, F value = 2.69, p = 0.103). **Healthy Drinking score increase** (0.88, F = 18.75, p<0.001 –v–control group score of 0.21, F value = 1.40, p = 0.239). **Physical Activity score increase** (0.30, F = 20.87, p<0.001 –v–control group score of 0.19, F value = 10.95, p<0.01). **Overall level of engagement in health- smart behaviors increase** (0.76, F = 26.47, p < .001 –v–Control group increase of 0.30, F value = 5.33, p = 0.022).	**Overall Low Risk of Bias**
**Sternberg et al., 2007 Multiple Countries, not fully listed Qualitative study**	Sternberg et al., Church/ church facilitated settings Combination of government and secular bodies Cardiovascular health risk Motivate adoption and maintenance of healthy behaviors.	Faith-based social groups, families, peers from religious community delivered individual, interpersonal and community level education and encouragement linking physical health to spiritual health, to adults from multiple countries.	✓		Indirect intervention consisting the social faith environment, religiosity and religious rituals	Behaviour modification relies on faith related factors including the spiritual and socio-cultural awareness of agents; making members of the faith community potentially effective agents.	**Overall high quality rating**
**Schoenthaler et al., 2018 USA (WHO Americas region) Randomised Controlled Trial**	TLC-MINT (Therapeutic Lifestyle change- plus Motivational Interviewing) Church/ Church premises Government Agency funded Hypertension Hypertension reduction (373) 172 intervention, 201 control	Trained professional researchers and church members trained as lay health advisors delivered individual and interpersonal level group counseling, therapeutic lifestyle training and motivational interviewing, to urban Black / African American adults across all income levels.	✓		Healthy Lifestyle Coaching, Counselling & Motivation Training	At 3 months: **Mean Arterial Pressure (MAP) decrease** (−8.5 mmHg 95%CI (−9.9 to −7.1)–V–control MAP decrease −7.2 95%CI (−7.8 to −6.6)). **Systolic Blood Pressure (SBP) decrease** (−13.2 in mmHg 95%CI (−14.6 to −11.8)–V–control group decrease of −10.1 95%CI (−10.7 to −9.5). **Diastolic Blood Pressure (DBP) decrease** −6.1mmHg 95%CI (−6.7 to −5.6)–V–control group decrease of −6.8 95%CI (−7.4 to −6.1). At 6 months: **MAP decrease** (−9.4 mmHg 95%CI (−11.4 to −7.4)–V–control group decrease of −7.3 95%CI (−8.2 to −6.5). **SBP decrease** (−14.6 mmHg 95%CI (−19.1 to −10.1)–V–control group decrease of −9.1 95%CI (−10.9 to −7.2). **SBP decrease** (−5.7 mmHg 95% CI (−6.0 to −5.5)–V–control group decrease of −6.4 95%CI (−6.7 to −6.2).	**Overall low risk of Bias**
**Liu et al., 2018 China (WHO Western Pacific region) Cohort study**	Routine Buddhist monk religious practice Buddhist Academy Governmental agency funded Association: Tibetan monk religious practice —— 594 participants	Experimental hypertension screening among Buddhist monks within the context of routine sedentary religious practice. Demonstration of the decreased odds for hypertension.		✓	Indirect intervention consisting the social faith environment, religiosity and religious rituals	Long hours of communal religious rituals and teaching in Tibetan Buddhist settings are associated with a decrease in odds for hypertension.	**Overall moderate risk of bias**
**Sørensen et al., 2011 Norway (WHO European Region) Cohort study**	The HUNT Study Church/churches Government agency funded Hypertension Religious attendance as hypertension intervention 35,964 individuals	Church clergy, social environment within churches and religious attendance as individual level intervention targeting Norwegian adults.		✓	Indirect intervention consisting the social faith environment, religiosity and religious rituals	Mean Systolic Blood Pressure (SBP) & mean Diastolic Blood Pressure (DBP) decreased with increasing Religious Attendance. Bivariate Associations between Religious Attendance and **DBP** (mmHg) showed **mean DBP** (and SD) of 71.1 (11.1), 70.9 (10.9), 71.9 (11.5) and 70.9 (11.7) for never attenders, 1–6 time per 6 month attenders, 1–3 times per month attenders and more than 3 times per month attending adults. p = 0.002. Religious Attendance and **SBP** (mmHg) showed **mean SBP** (and SD) of 127.6 (19.5), 127.8 (19.7), 134.5 (21.7) and 131.3 (21.2) for never attenders, 1–6 time per 6 month attenders, 1–3 times per month attenders and more than 3 times per month attending adults. p < 0.001.	**Overall low risk of bias**
**Kinard, 2016 USA (WHO Americas region) Controlled before- and after study**	The My Sister’s Keeper Project Church Church/churches Government agency funding Hypertension and body weight Reduction of body weight and blood pressure. (19) 4 intervention, 15 control	Theologically trained ministers and trained health coaches delivered individual and interpersonal level spiritually based nutrition and physical activity education, bible verses and prayers to urban Black / African American adults across all income groups.	✓		Combination of Nutrition & Exercise / Physical Activity	**Mean Systolic Blood Pressure (SBP) decrease** (0.66 mmHg, Z value = 0.00, p = 0.999,–V–control group mean reduction of 3.0 mm, Z value = -0.734, p = 0.463). **No change in mean Diastolic Blood Pressure (DBP)** (0mmHg, Z = 0.00, p = 0.999 –V–control group reduction of 4.71 mmHg, Z value = -1.185, p = 0.236). **Mean Body Weight increase** of 1.0 lbs, Z value = -.816, p = .414, -V- control group mean weight reduction of 1.3 lbs, Z value = -.676, p = 0.499). Z value = Z value in Wilcoxon text	**Overall moderate risk of bias**
**Abbot, 2015 USA (WHO Americas region) Controlled before- and after study**	With Every Heartbeat is Life Church/churches Government agency funding Cardiovascular risk Increase cardiovascular health knowledge (229) 114 intervention, 115 control	Trained health educators delivered individual and interpersonal level health promotion to rural Black / African American adults.	✓		Healthy Lifestyle Coaching, Counselling & Motivation Training	Greater improvements in **cardiovascular health habits (*p* < .01) and health knowledge (*p* < .01)** compared to the control group on both analyses of repeated measures and gain scores. The intervention positively affected **intentions to consume more fruits and vegetables** (*p* = 0.01)**, reduce dietary fat** (*p* = 0.01), but did not the **intentions to increase exercise**. The intervention positively affected the **norms and attitudes** of the participants on consuming more produce (*p* = 0.01)), reducing dietary fat (*p* = 0.04), but had no effect on their attitudes and norms on participating in exercising. The intervention had positive effects on participants in increasing their **perceived behavioural control/enhancing their self-efficacy** (*p* < 0.01) for increasing their **fruit and vegetable intake** (*p* < 0.01), reducing their **dietary fat intake** (*p* = 0.03), and increasing their **exercise** (*p* < 0.01).	**Overall moderate risk of bias**
**Taylor, 2011 USA (WHO Americas region) Case series (uncontrolled longitudinal) study**	Way of Faith (WOF) project Church/churches Non Profit Organisation Funding Cardiovascular disease Health promotion to reduce obesity, hypertension and high blood sugar 15 participants	Qualified wellness counsellors, nurses and clergy delivered individual, interpersonal and community level eating habits and exercise workshops to urban adults of all ethnicities.	✓		Combination of Nutrition & Exercise / Physical Activity	No significant change, with 1.7 mmHg **Mean DBP** decrease from 86.3 to 84.6 mmHg. **Mean pulse rate** increase of 3.5 beats per minute from 70.9 to 74.4. **Mean weight** increase of 1.18 kg from 146.83 to 148.02. **Mean blood sugar** 5.72 mg/dL increase from 100.57 to 106.29. At 6 months, significant change in **attitudes and behavioral understanding**: 80% of participants had started exercising, a 60% increase. 90% of participants reported a change of their eating habits. 100% of participants reported greater responsibility in taking care of their bodies.	**Overall moderate risk of bias**
**Daye, 2019 USA (WHO Americas region) Controlled before and after study**	Faith-based health devotional Churches/private residences Non Profit Organisation Funding Hypertension Increase knowledge on hypertension and hypertension prevention 100 participants	Implementation of faith-based health devotional on rural and urban Black / African American adult attenders of faith institutions.	✓		Healthy Lifestyle Coaching, Counselling & Motivation Training	Increase in **general knowledge about high blood pressure and its prevention,** indicated in statistically significant increase from 8.28 to 9.09 of mean High Blood Pressure Prevention IQ Quiz- 0.81, 95% CI (1.02–0.60), df = 99, p < .000. Non-significant increased **understanding of the negative consequences of poor blood pressure control** indicated by 3.25 to 3.40 change, a 0.15 increase in Consequences scores 0.15, 95% CI (0.002–0.298), df = 99, p < 0.24. Significant increase in **perceived personal control of blood pressure** indicated by 4.11 to 4.30 change, a 0.19 increase in Personal Control scores 0.19, 95% CI (0.079–0.311), df = 99, p < 0.0005.	**Overall moderate risk of bias**
**Dodani et al., 2014 USA (WHO Americas region) Prospective uncontrolled longitudinal study**	Healthy Eating and Living Spiritually (HEALS) Church/churches Funded by a publicly funded university Hypertension Blood Pressure reduction 34 participants	Pastors and trained church health advisors delivered at the individual and interpersonal level, socio-culturally informed dietary modification, increased physical activity and healthy behavioral change to rural and urban Black / African American adults across all income levels	✓		Combination of Nutrition & Exercise / Physical Activity	**Reduction** in Mean **Systolic Blood Pressure** (**SBP)** of 22 mmHg (p < 0.001). **Reduction** in Mean **Diastolic Blood Pressure** (**DBP)** of 6.5 mmHg (p = 0.0048) A **mean weight** reduction of 3.11 kg (p < 0.0001).	**Overall moderate risk of bias**
**Dodani et al., 2013 USA (WHO Americas region) Prospective uncontrolled longitudinal study**	Healthy Eating and Living Spiritually (HEALS) Church/churches Funded by a publicly funded university Risk of stroke and hypertension Blood Pressure reduction 31 participants	Pastors and trained church health advisors delivered at the individual and interpersonal level, socio-culturally informed dietary modification, increased physical activity and healthy behavioral change to rural and urban Black / African American adults across all income levels	✓		Combination of Nutrition & Exercise / Physical Activity	Reduction in Mean **Systolic Blood Pressure** (**SBP)** of 13.64 mmHg (p = 0.005). Mean **Diastolic Blood Pressure** (**DBP)** of 6.12 mmHg (p = 0.01).	**Overall moderate risk of bias**
**Dodani et al., 2015 USA (WHO Americas region) Prospective uncontrolled longitudinal study**	Healthy Eating and Living Spiritually (HEALS) Church/churches Funded by a publicly funded university Risk of stroke and hypertension Blood Pressure reduction 36 participants	Pastors and trained church health advisors delivered at the individual and interpersonal level, socio-culturally informed dietary modification, increased physical activity and healthy behavioral change to rural and urban Black / African American adults across all income levels.	✓		Combination of Nutrition & Exercise / Physical Activity	Reduction of 6.72 mmHg in Mean **Systolic Blood Pressure** (**SBP)** (p = 0.0425) and 4.0 mmHg in **Diastolic Blood Pressure** (**DBP)** (p = 0.0073). Mean **weight** reduction of 1.75kg (p = 0.0023).	**Overall moderate risk of bias**
**Baig et al., 2015 USA (WHO Americas region) Randomised Controlled Trial**	Picture Good Health Church/churches Government agency funded Cardiovascular risk factors Self management of cardiovascular risk factors (100) 50 intervention, 50 control	Trained lay church leaders delivered individual and interpersonal level cardiovascular self-management classes to urban American Latino adults of all income levels.	✓		Healthy Lifestyle Coaching, Counselling & Motivation Training	3 Months: Intervention group **Mean Systolic BP decrease** of −2.72 95%CI (−7.19 to 1.74). Control group Mean SBP increase of 1.42 mmHg 95% CI (−3.29 to 6.13). 6 months: Intervention group **Mean SBP decrease** of −4.62 95%CI (−9.14 to −0.09). Control group Mean SBP increase of 0.6 mmHg 95% CI (−3.85 to 5.04). 3 months: Intervention group **Mean DBP decrease**, -4.07mmHg 95% CI (-7.19 to -0.94). Control group Mean DBP increase of 1.78 mmHg 95% CI (−1.5 to 5.06). 6 months: Intervention group **Mean DBP decrease**, -3.14mmHg 95% CI (-6.59 to -0.31). Control group Mean DBP increase of 0.91 mmHg 95% CI (−2.5 to 4.33). 6 months: Intervention group **Mean Glycosylated haemoglobin** (%) decrease of -0.27 95%CI (-0.7 to 0.14) in 3 months and -0.27 95%CI (-0.81 to 0.28). 6 months: Control group Mean **Glycosylated haemoglobin (%) decrease** of -0.05 95%CI (-0.59, 0.48) in 6 months and -0.05 95%CI (-0.59, 0.48). 6 months: Intervention group **Mean Low Density Lipoprotein (mg/dL) decrease** of -0.16 95%CI (-6.61, 6.3) in 3 months and -4.94 95%CI (-13.64, 3.77). 6 months: Control group **Mean Low Density Lipoprotein (mg/dL) increase** of 0.97 95%CI (-5.78, 7.72) in 3 months and 2.55 95%CI (-6.04, 11.13). 6 months: Intervention group **Mean Waist Circumference (cm) decrease** of -0.93 95%CI (-2.63, 0.76) in 3 months and -9.8 95%CI (-2.83, 0.88). 6 months: Control group Mean Waist Circumference (cm) decrease of -0.22 95%CI (-1.99, 1.55) in 3 months and -0.51 95%CI (-2.34, 1.32).	**Overall some concerns risk of Bias**
**Draper et al., 2019 South Africa (WHO Africa region) Prospective uncontrolled longitudinal study**	Health through Faith [Impilo neZenkolo] Church/churches Government agency funding Cardiovascular risk Healthy lifestyle to reduce cardiovascular risk 84 participants	Church leaders and members delivered individual and interpersonal lifestyle teaching sessions to low income rural and urban Black / African adults.	✓		Healthy Lifestyle Coaching, Counselling & Motivation Training	**Mean Systolic BP reduction** of 1 mmHg from 123 95%CI (107, 132) to 122 95%CI (116, 134), p = 0.085, z = -1.721. **Mean Diastolic BP increase** of 3 mmHg from 81, 95%CI (72, 86) to 84, 95%CI (74, 92), p = 0.451, z = -0.753. **Mean weight reduction** of 2.2kg 80.5 ± 20.1 to 78.3 ± 19.1, p = 0.010 **Mean BMI reduction** of 0.8 kg/m^2^ from 29.9 ± 7.4 to 29.1 ± 7.1, p = 0.01. **Mean waist circumference** decrease of 4.1 cm from 92.3 ± 17.4 to 88.2 ± 15.9, p = 0.02. Other improvements in self reported measures of **dietary habits**, and **self-reported health status**, **psychological distress and self-esteem scores**.	**Overall moderate risk of bias**
**Lee et al., 2018 USA (WHO Americas region) Qualitative study**	Intensive peer group intervention Churches/community Government agency funding Cardiovascular disease risk Reduce risks for heart disease	Male church members and peer facilitators delivered individual and interpersonal level intensive heart disease knowledge, awareness and support sessions to urban Black / African American adults.	✓		Healthy Lifestyle Coaching, Counselling & Motivation Training	Intensive peer group intervention was effective to promote cardiovascular health beneficial behaviour change through: Enhancing access to health behavior information and resources; Practicing and applying problem-solving skills with group feedback and support; Discussing health behavior challenges and barriers; Sharing health behavior changes; Sharing perceived health outcome improvements and benefits; The feeling of belonging and being cared for; Addressing health of family and community.	**Overall high quality rating**
**Benjamin, 2017 USA (WHO Americas region) Uncontrolled before and after study**	Smart Self Management intervention Churches/ homes Non Profit Organisation Hypertension Increase hypertension knowledge and self management 23 participants	Faith institution participating rural and urban Black Haitian American adults across all income levels delivered individual and interpersonal level hypertension education informed and culturally appropriate self-created SMART management.	✓		Healthy Lifestyle Coaching, Counselling & Motivation Training	Increases in knowledge gained and ability to create and maintain SMART goals: Significant increase in **Basics of hypertension** scores from a mean pre-test 55% to mean post-test of 85%. 30%, 95% CI (0.23–0.37). T = 9.376, p < 0.001. Increase in **Dietary modifications & BP management** scores from a mean pre-test 76% to mean post-test of 89.3%. 11%, 95% CI (0.1–0.32). T = 1.2301, p = 0.2584. Significant increase in **Physical activity & BP** scores from a mean pre-test 64% to mean post-test of 87%. 24%, 95% CI (0.13–0.35). T = 5.0992, p = <0.001. Significant increase in **medication management & symptom recognition** scores of 31%, 95% CI (0.21–0.42). T = 6.74, p = <0.001.	**Overall serious risk of bias**
**Spell-LeSane, 2016 USA (WHO Americas region) Uncontrolled before and after study**	I am Working on My Heart: A Cardiovascular Disease Awareness Program Church/churches Government agency funding Cardiovascular risk Increase cardiovascular disease knowledge, awareness and motivation 137 participants	Registered nurses delivered individual and interpersonal level cardiovascular disease knowledge, awareness learning and motivation sessions to rural and urban Black / African American adult women across all income levels	✓		Healthy Lifestyle Coaching, Counselling & Motivation Training	**Participant awareness** of cardiovascular disease as leading cause of death significantly increased 27% from 63% to 90% (p = <0.001). **Participant knowledge of Cardiovascular disease improved with the** proportion of very well informed women increasing 28% from 3% to 31% (p = <0.002). **Participant engagement in Physical activity improved.** 27% of previously not active participants increased the frequency and intensity of physical activity significantly (p <0.0001). 18% of participants who pre-intervention engaged sub-optimally in physical activity (PA) increased their PA to meet the guidelines. 42% increase in physical activity of women with optimal pre-intervention physical activity levels.	**Overall low risk of bias**
**White, 2018 USA (WHO Americas region) Uncontrolled before and after study**	Check. Change. Control. Churches/ homes No Funding identified Hypertension Reduce Blood Pressure 23 participants	Volunteer hypertensive Black / African American adults across all income levels self-delivered an American Heart Association (AHA) self management programme	✓		Healthy Lifestyle Coaching, Counselling & Motivation Training	**Minimal statistically non-significant Systolic Blood Pressure (SBP) and Diastolic Blood Pressure (DBP) reduction:** Mean SBP decrease of 3.09mmHg, 95%CI (0–6.558), T = 1.844, p = 0.079. Mean DBP decrease of 2.26mmHg, 95%CI (-0.459–5.59), T = 0.724, p = 0.099.	**Overall moderate risk of bias**
**Bittman et al., 2020 USA (WHO Americas region) Randomised Controlled Trial**	Gospel Music intervention Churches Nongovernmental Organisation funding Hypertension risk Improve engagement in cardiovascular risk reduction (71) 36 intervention, 35 control	A researcher and a team of local congregation derived cardiologist, musician facilitators, registered nurses and volunteer health workers, delivered Gospel music driven engagement in cardiovascular risk reduction programme to urban Black / African American adults across all income levels	✓		Healthy Lifestyle Coaching, Counselling & Motivation Training	**Statistically non-significant reduction of Systolic Blood Pressure (SBP) (p = 0.54) and Diastolic Blood Pressure (DBP) (p = 0.41).** SBP Intervention group Pre-intervention 140.99 95%CI (136.12 to 145.85), post-intervention 133.97 (129.28 to 138.66); Control group pre-intervention 138.48 (312.65 to 144.31), post-intervention 130.10 (124.48 to 135.73). DBP Intervention group Pre-intervention 82.88 95%CI (79.17 to 86.60), post-intervention 82.20 95%CI (78.49 to 85.92), Control group pre-intervention 80.33 95%CI (75.88 to 84.78), post-intervention 76.29 95%CI (71.84 to 80.75). Reduction of weight, hip and waist circumference. No significant between group effect for weight, F(1,41) = 0.22, p = 0.64: Intervention group pre-intervention 200.29 95%CI (185.09, 215.48), post-intervention 196.57 95%CI (182.37, 210.77). Control group pre-intervention 174.07 95%CI (153.30, 194.83), post-intervention 178.40 95%CI (159.00, 197.80). No significant between group effect for Hip circumference, F(1,41) = 0.16, p = 0.70: Intervention group pre-intervention 48.07 95%CI (45.99, 501.5), post-intervention 48.21 95%CI (46.07, 50.37). Control group pre intervention 43.73 (40.89, 46.58), post-intervention 44.87 (41.92, 47.82).	**Overall high risk of Bias**
**Ray, 2003 USA (WHO Americas region) Non-Randomised Controlled Trial**	Church-based nutrition intervention Churches No Funding identified Hypertension and hypertension risk Improve health by weight loss and Blood Pressure reduction (31) 19 intervention, 12 control	Three nurses and a dietician from the local community delivered individual and interpersonal Scripture supported, culturally competent nutrition education workshops to rural and urban Black / African American adults across all income levels	✓		Nutrition	**Mean Systolic Blood Pressure (SBP) decrease** of 7.2 mmHg–v–control group mean SBP increase of 2.78mmHg (p = 0.935). **Mean Diastolic Blood Pressure (DBP) decrease** of 6.93 mmHg–v–control group mean DBP increase of 2.33mmHg (p = 0.961). Significant **reduction in the weight** of participants in the treatment group, F(3,12) = 5.29, p<0.05 compared to non-significant increase in weight of the participants in the control group. **Increased awareness** about blood pressure, p<0.05, in the intervention group.	**Overall low risk of bias**
**Wiist et al., 1990 USA (WHO Americas region) Randomised Controlled Trial**	Pilot cholesterol education programme Churches Government agency funding Cardiovascular risk Cardiovascular screening and nutrition education (348) 174 intervention, 174 control	Church leaders and volunteer health professional members delivered individual and interpersonal cardiovascular screening and nutrition education to rural and urban Black / African American adults across all income levels	✓	✓	Nutrition	**Cholesterol levels decrease** from 233.9mg/dl to 210.4mg/dl (p<0.0001) compared to control group Cholesterol levels decrease from 241.5mg/dl to 202.9mg/dl (p<0.0001). No statistically significant differences between the intervention and control group baseline or follow-up levels.	**Overall some concerns risk of Bias**
**Yanek et al., 2001 USA (WHO Americas region) Randomised Controlled Trial**	Project Joy Churches Government agency funding Cardiovascular risk Improve cardiovascular risk profile (529) 455 intervention, 74 control	Lay church leaders and health educators delivered individual, interpersonal and community level cardiovascular risk profile improving nutrition and physical activity education to urban Black / African American adult women across all income levels	✓		Combination of Nutrition & Exercise / Physical Activity	**Mean Systolic Blood Pressure (SBP) decrease** of 1.6 mmHg–V–control group mean SBP decrease of 0.95, p = 0.47. **Mean Diastolic Blood Pressure (DBP) decrease** of 0.36 mmHg–V–control group mean SBP increase of 0.22, p = 0.49. In the top 10% cohort by weight loss: **Mean SBP decrease** of 8.1 mmHg (p = 0.0005)–V–control group mean SBP decrease of 3.3 mmHg (p = 0.5688). **Mean DBP decrease** of 4.4 mmHg (p = 0.0004)–V–control group mean DBP increase of 0.8 mmHg (p = 0.7805). **Mean body weight decrease** of 0.5kg–V–control group mean weight increase of 0.38kg (p = 0.0008). **Mean Body Mass Index (BMI) decrease** of 0.17 kg/m^2^ –v–control group BMI increase of 0.14 kg/m^2^ (p = 0.0012). **Mean daily Sodium intake decrease** of 145mg/day–v–control group decrease of 8 mg/day, p = 0.0167.	**Overall some concerns risk of Bias**
**Smith et al., 1997 USA (WHO Americas region) Uncontrolled before and after study**	Church-based education and support programme Churches Government agency funding Hypertension Hypertension reduction 97 participants	Nurses trained as church health educators and lay church members trained as organizers and facilitators, delivered individual and interpersonal level hypertension education and practical support to urban Black / African American adults across all income levels	✓		Healthy Lifestyle Coaching, Counselling & Motivation Training	**Mean Arterial Pressure decrease** (p ≤ 0.0001, F = 17.80, df = 1,86) **Systolic Blood Pressure decrease** (p ≤ 0.0001, F = 18.35, df = 1,91) **Diastolic Blood Pressure decrease** (p ≤ 0.008, F = 17.48, df = 1,91) **Hypertension knowledge** scores significantly increased and remained higher than baseline at 3 months after end of intervention (p ≤ 0.0001; F = 95.08; df = 1,79).	**Overall moderate risk of bias**
**Oexmann et al., 2001 USA (WHO Americas region) Uncontrolled before and after study**	Lighten Up lifestyle programme Churches Nongovernmental Organisation funding Cardiovascular risk Reduce cardiovascular risk 381 participants	Volunteer lay church members trained as health educators delivered individual and interpersonal level lifestyle modification education to urban White and Black / African American adults across all income levels	✓		Healthy Lifestyle Coaching, Counselling & Motivation Training	Post 0–5 session attendance: **Systolic Blood Pressure (SBP) decrease** of 1.2 ± 1.2 (mmHg), p>0.5 for Blacks; decrease of 5.6 ± 1.7(mmHg), p≤ 0.01 for Whites. **Diastolic Blood Pressure (DBP) decrease** of 0.2 ± 0.9(mmHg), p>0.5 for Blacks; decrease of 2.2(mmHg) ± 1.0, p≤ 0.05 for Whites. Post 6–8 sessions attendance: **SBP decrease** (mmHg) of 6.3 ± 1.2, p>0.5 for Blacks; decrease of 8.1 ± 1.7, p≤ 0.01 for Whites. **DBP decrease** (mmHg) of 2.5 ± 0.8, p ≤ 0.01 for Blacks; **decrease** of 3.6 ± 1.2, p≤ 0.01 for Whites. Post 0–5 sessions attendance: **Body weight decrease** (pounds) of 2.7 ± 0.4, p < 0.001for Blacks; 3.6 ± 0.8, p < 0.001 for Whites. **Total cholesterol increase** (mg/dl) of 1.0 ± 2.6, p>0.5 for Blacks; and a **decrease** of 13.2 ± 4.0, p≤ 0.05 for Whites. Post 6–8 sessions attendance: **Body weight decrease** (pounds) of 3.5 ± 0.4, p < 0.001for Blacks; 6.3 ± 0.6, p < 0.001decrease for Whites. **Total cholesterol decrease** (mg/dl) of 4.9 ± 2.3, p<0.05 for Blacks; decrease of 14.5 ± 2.9, p≤ 0.001 for White participants.	**Overall moderate risk of bias**

### Risk of bias assessment

**Fig 2:**
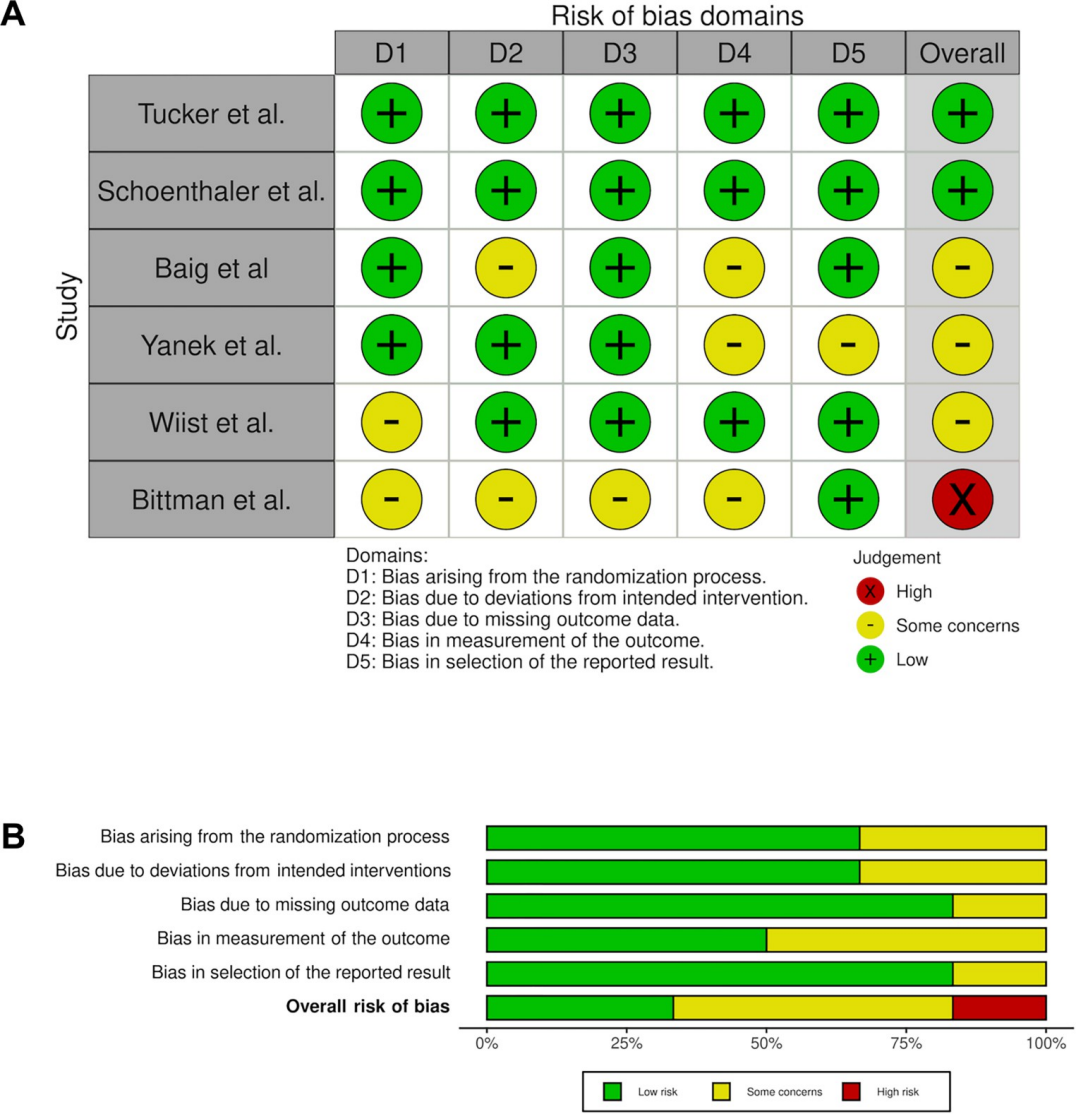
Bias assessment of included randomised studies.

**Fig 3:**
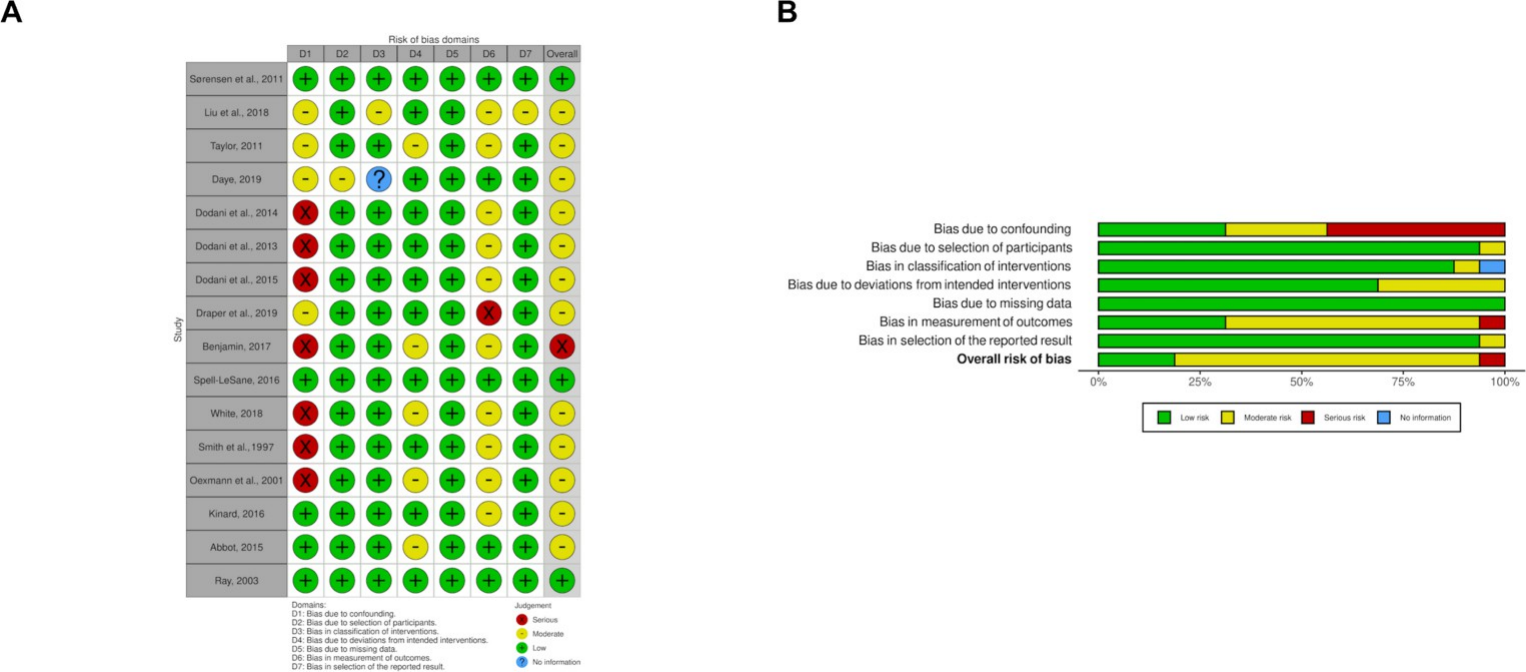
Bias assessment of the included non-randomised studies.

The qualitative studies received a high overall quality rating (Table 5). Of the 6 randomised studies, two were rated low risk of bias, 3 with some concerns, and one high bias risk. Three nonrandomised studies were rated low risk and 12 rated moderate risk of bias. One [59] was adjudged with serious risk of bias due to potential confounding factors, although four sub-domains were low risk and two sub-domains moderate risk. The confounding element of nonrandomised studies was the most frequently adjudged at serious risk—perhaps a reflection of the multiple, complex and interdependent nature of cardiovascular risks (Figs 2A, 2B, 3A and 3B).

**Table 5:** Summary of the risk of bias assessments in the included qualitative studies, using the Qualitative Assessment and Review Instrument (QARI) quality assessment tool with criteria rated as yes, no or unclear.

Study	Risk of Bias Criteria										Overall Quality Rating
	1	2	3	4	5	6	7	8	9	10	
Sternberg et al., 2007	Unclear	Yes	Yes	Yes	Yes	No	No	Unclear	Unclear	Yes	**High**
Lee et al., 2018	Yes	Yes	Yes	Yes	Yes	Unclear	Yes	Yes	Yes	Yes	**High**

## Faith institution roles

Faith institution roles constituted a variety of promotion of, provision for and persuasion of participant engagement in cardiovascular activities. Twenty-two studies incorporated cardiovascular health promotion and education roles. Two cohort studies did not, being mainly blood pressure measurement studies [57, 58]. One study combined cardiovascular health promotion and education roles with blood pressure measurement [60]. Altogether, the 24 studies reported 19 different types of relevant outcomes.

Blood pressures were reported by 17 studies; and mean arterial blood pressure outcomes reported by two [34, 61]. Secondary outcome measures (risk modifying elements) are diverse, mostly quantitative and include body weight, waist circumference and hip circumference measurements, Body Mass Index, nutrition literacy scores, physical activity, healthy behaviour engagement scores, healthy cardiovascular knowledge scores, blood sugar measurements, serum glycosylated haemoglobin measurements, serum Low Density Lipoprotein measurements, serum cholesterol measurements and dietary sodium.

Some secondary outcomes were more complex to measure, including adoption of healthier diets, nutrition label literacy awareness, engagement in health—smart behaviors, increased hypertension monitoring and engagement with healthcare system. Process indicator outcomes were reported for studies and include intervention uptake levels, extent of participation, intervention acceptability and participant satisfaction [56, 62–64].

Four studies incorporated and reported on personal psychological effectiveness [59, 61, 65, 66]. A single study reported on personal control and understanding of consequences as outcome measures [67]. One study reported faith related factors, such as targeted training with religious content, pastoral leadership and sociocultural awareness of educators being influential in personal effectiveness for behaviour modification [55]. A single study addressed nutrition label literacy promoting activities contributing to cardiovascular health promotion [68].

The biological markers targeted in education and health promotion include blood sugar, glycosylated haemoglobin, cholesterol and low-density lipoprotein levels. A single study reported blood sugar levels as part of the intervention outcomes [69]. Lowering serum glycosylated haemoglobin, a marker of the effectiveness of diabetes control, was reported by a single study [62]. Two studies directly reported the outcomes of serum cholesterol levels [60, 70]. A single study reported the closely related outcome of Low Density Lipoproteins, an indirect marker of the risk of coronary heart disease [62].

Alteration of biological markers involves manipulation of energy consumption and expenditure, notably through nutrition and exercise. Two studies directly reported the outcomes of healthy eating or healthy eating habits [68, 69], while a single study captured the same in participant satisfaction outcomes [62]. Closely related is the promotion of healthy drinking habits, which was reported by one study [68].

Motivation or intention for dietary change, usually toward increasing fibre, fruit and vegetable consumption and reducing dietary fat is part of the activities contributing to the health promotion. Four studies reported the outcome of dietary change behaviour or intention [56, 59, 62, 65].

Activities targeting attitudes toward exercise or physical activity contribute to health promotion roles. Five studies incorporate attitudes to or uptake of exercise or general physical activity, and reported them [59, 65, 66, 68, 69]. A single study used measures that influence self reported psychological wellness or self-esteem [56].

### Quantitative synthesis

#### Primary outcomes

The primary outcomes, Systolic Blood Pressure (SBP) and Diastolic Blood Pressure (DBP) indicate the estimate and direction of effect of the interventions.

#### Meta-analyses of primary outcomes in randomised studies

**Fig 4:**
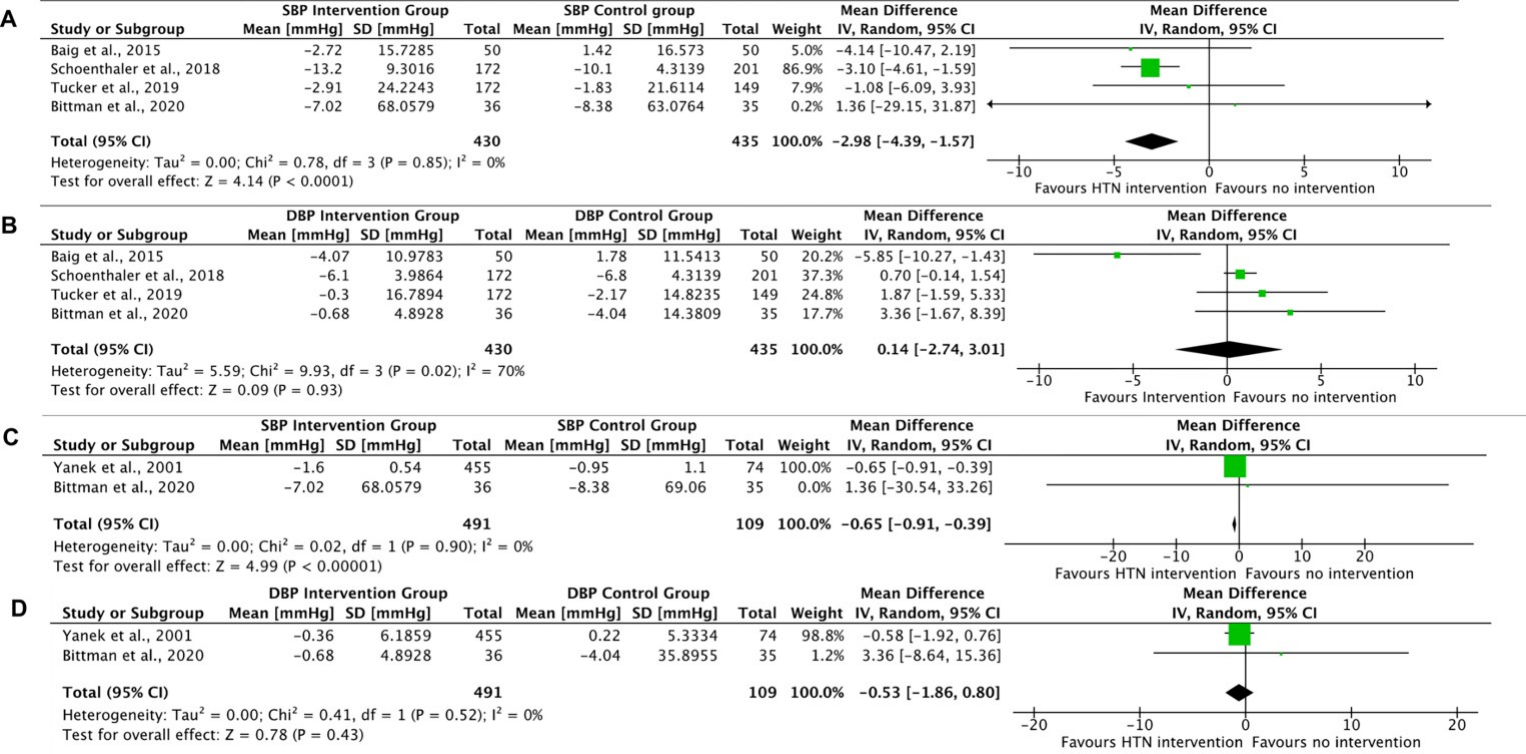
Forest plot of meta-analysis of change in systolic and diastolic blood pressures 3 and 12 months post hypertension intervention.

Only four RCTs with 865 participants could contribute to the meta-analysis of blood pressure outcomes at 3 months with one study intervention outcomes unavailable before 12 months follow-up [71], and the other having no blood pressure outcomes [60]. Using a random effects model, the overall pooled estimate for mean reduction of systolic BP three months after the interventions was significant at 2.98 mmHg (95%CI -4.39 to -1.57), compared to non-participation in hypertension intervention. Heterogeneity was measured as I^2^ 0% (Fig 4A). The overall pooled estimate shows a non-significant mean increase of 0.14 mmHg (95%CI -2.74 to +3.01) in the diastolic BP three months after the interventions. Heterogeneity, I^2^ = 70% (Fig 4B).

Meta analysis was conducted on the two RCTs (including 600 participants) that recorded systolic and diastolic blood pressure outcome measures a year post intervention. Using a random effects model, showed an overall pooled estimate of significant mean reduction of 0.65 mmHg (95%CI -0.91 to -0.39) in systolic BP twelve months after the interventions. Heterogeneity, I^2^ = 0% (Fig 4C). Using a random effects model, showed an overall pooled estimate of non-significant mean reduction of 0.53 mmHg (95%CI -1.86 to 0.80) in diastolic BP twelve months after the interventions. Heterogeneity, I^2^ = 0% (Fig 4D).

#### Meta-analyses of secondary outcomes in randomised studies

**Fig 5:**
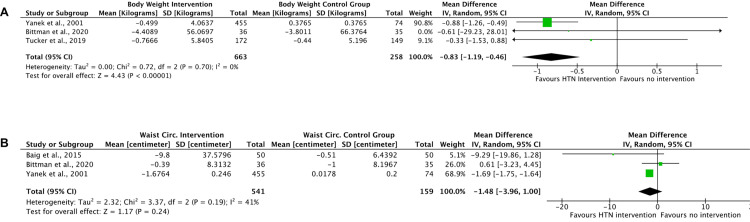
Forest plot of meta-analysis of body weight change and waist circumference change at end of intervention.

Meta-analyses of the RCTs that measured body weight and waist circumferences changes indicates an overall small but beneficial intervention effect at the end of the interventions (3 months for Tucker et al. and Baig et al., 12 months for Yanek et al. and Bittman et al.): significant mean weight reduction of 0.83kg (95% CI -1.19 to -0.46), I^2^ = 0% and non-significant mean waist circumference reduction of 1.48cm (95% CI -3.96 to +1.00), I^2^ = 41% (Fig 5A and 5B).

#### Primary outcomes in Non-randomised studies

Rather than single or summary estimate of effects, an indication is presented of the effect of faith institution facilitated activities on the direction of effect of the available outcomes.

With the exception of a single study where post-intervention diastolic blood pressures increased [56], systolic and diastolic blood pressures decreased. Nearly half of the reductions were statistically significant (Table 6). One study reported a mixed picture of diastolic blood pressure reduction, with significant diastolic blood pressure reductions for white and non-significant diastolic blood pressure reductions for Black participants [70].

**Table 6:** Summary non-aggregated overview of direction of blood pressure change following interventions reported in nonrandomised studies.

Study	Systolic Blood Pressure	Diastolic Blood Pressure
	(Significance)	(Significance)
**Taylor, 2011**	**Reduction^†^**	**Reduction^†^**
	(p = 0.30)	(p = 0.38)
**Kinard, 2016**	**Reduction^†^**	**Reduction^†^**
	(p = 0.99)	(p = 0.99)
**Dodani et al., 2014**	Reduction*	Reduction*
	(P = 0.001)	(P = 0.0048)
**Dodani et al., 2013**	Reduction*	Reduction*
	(P = 0.005)	(P = 0.01)
**Dodani et al., 2015**	Reduction*	Reduction*
	(P = 0.0425)	(P = 0.0073)
**Draper et al., 2019**	**Reduction^†^**	**Increase^∂^**
	(p = 0.085)	(p = 0.451)
**White, 2018**	**Reduction^†^**	**Reduction^†^**
	(p = 0.079)	(p = 0.099)
**Smith et al., 1997**	Reduction*	Reduction*
	(p = 0.0001)	(P = 0.008)
**Oexmann et al., 2001**	**Reduction**	**Reduction**
	(Mixed) Blacks p = 0.5 Whites p = 0.01	(Mixed) Blacks p = 0.01 Whites p = 0.01
**Ray, 2003**	**Reduction^†^**	**Reduction^†^**
	p = 0.935	p = 0.961

Reduction* = P<0.05; Reduction^†^ = P>0.05; Increase^∂^ = P>0.05; Mixed = significant reduction for white and non-significant reduction for Black participants.

### Secondary outcomes in nonrandomised studies

Four of the included nonrandomised studies reported no secondary outcome measures, while 12 studies recorded a large number of distinct outcomes. These included mean weight; blood sugar; knowledge of hypertension or cardiovascular disease and health; attitudes to diet and exercise; hypertension or cardiovascular disease awareness; personal control; total cholesterol; Body Mass Index; waist circumference and physical activity, among others. A large diversity of methodologies was employed in the studies, rendering aggregation of secondary outcome measures difficult. However individually the distinct secondary outcomes generally indicate beneficial post-intervention effects for hypertension and cardiovascular health (Table 7).

**Table 7:** Overview of distinct secondary outcomes in nonrandomised studies, and their impact on hypertension and cardiovascular health.

Study/ Intervention	Distinct Secondary Outcome	Intervention impact on Hypertension or Cardiovascular Health, based on single distinct secondary outcome as a risk or protective factor		
		Significantly beneficial	Non-significantly beneficial	Not beneficial
Kinard (2016) My Sister’s Keeper Project	Weight			Intervention group non-significant mean weight increase (p>0.05). Control group non-significant weight reduction (p>0.05).
Ray (2003) Church-based nutrition intervention Intervention	Weight	Intervention group significant (p<0.05) mean weight reduction. Control group—non-significant mean weight increase (p>0.05).		
Dodani (2014) Healthy Eating and Living Spiritually (HEALS) Dodani (2015) Healthy Eating and Living Spiritually (HEALS) Draper (2019) Health through Faith [Impilo neZenkolo] Oexmann (2001) Lighten Up lifestyle programme	Weight	Significant mean weight reduction: Dodani (2014) p < 0.05; Dodani (2015) p = 0.0023; Draper (2019) p = 0.010 Oexmann (2001) p < 0.001		
Taylor (2011) Way of Faith (WOF) project	Weight			Small non-significant weight increase
Daye (2019) Faith-based health devotional intervention	Knowledge	Significant increase in Hypertension Prevention IQ Quiz scores p < .0001		
Benjamin (2017) Smart Self Management intervention	Knowledge	Significant increase in Basics of Hypertension Scores p < 0.001.		
Spell-LeSane (2016) I am Working on My Heart Cardiovascular Disease Awareness intervention	Knowledge	Significant increase in the proportion of well informed participants in knowledge of cardiovascular disease. p <0.002		
Smith (1997) Church-based education and support intervention	Knowledge	Significant post intervention hypertension knowledge scores, p ≤ 0.0001		
Abbot (2015) ‘With Every Heartbeat is Life’ intervention,	Knowledge	Significant increase (*p* < .01) in cardiovascular health knowledge scores of intervention participants compared to the control group		
Ray (2003) Church-based nutrition intervention Intervention	Blood pressure awareness	Significant increase in blood pressure awareness index, p<0.05		
Spell-LeSane (2016) I am Working on My Heart Cardiovascular Disease Awareness intervention	Awareness as leading cause of death	Significant increase in awareness of hypertension and cardiovascular disease as leading cause of death, p = <0.001		
Daye (2019) Faith-based health devotional intervention	Perceived personal control	Significant increase in Personal Control scores, p < 0.0005		
Abbott (2015) ‘With Every Heartbeat is Life’ intervention,	Perceived behavioural control to enhance self-efficacy	Significant increase in self-efficacy scores to increase fruit and vegetable intake, *p* < 0.01. Significant increase in self-efficacy scores to reduce dietary fat intake, *p* = 0.03. Significant increase in self-efficacy scores to increase exercise, *p* < 0.01.		
Spell-LeSane (2016) I am Working on My Heart Cardiovascular Disease Awareness intervention	Engagement in physical activity	Significant increase in frequency and intensity (27% of pre-intervention inactive participants, p <0.0001) Increase in physical activity (18% of pre intervention optimally exercising participants) Increase in physical activity (42% of women with adequate pre-intervention physical activity levels)		
Benjamin (2017) ‘Smart Self Management’ intervention	Engagement in physical activity	Significant increase in pre-test to post-test physical activity scores, p <0.001.		
Oexmann (2001) ‘Lighten Up lifestyle programme’	Total cholesterol	Significant total cholesterol reduction, p<0.05.		
Abbott (2015) ‘With Every Heartbeat is Life’	Attitudes to fruits/vegetables, dietary fat, and exercise	Significant increased fruits and vegetables consumption, *p* < 0.01. Significant reduction in dietary fat, *p* = 0.03.		No change in attitude toward exercise or physical activity
Taylor (2011) ‘Way of Faith (WOF) project’ intervention	Attitudes to taking care of self	Post intervention greater self care responsibility: An additional 60% of participants started exercising. 90% of participants positively changed their eating habits		
Taylor (2011) ‘Way of Faith (WOF) project’ intervention	Blood sugar			Post-intervention Blood Sugar increase
Draper (2019) Health through Faith [Impilo neZenkolo]	Body Mass Index	Significant post-intervention Body Mass Index reduction, p = 0.01.		
Daye (2019) Faith-based health devotional intervention	Understanding of the negative consequences of poor blood pressure control		A non-significant increase in understanding of the negative consequences of poor blood pressure control, p < 0.24.	
Abbott (2015) ‘With Every Heartbeat is Life’	Intention to change fruits / vegetables, dietary fat, and exercise habits	Significant increase in scores for intention to consume more fruits and vegetables, *p* < .01. Significant increase in scores for intention to reduce dietary fat, *p* < .01.		No change in scores for intention to increase exercise
Benjamin (2017) ‘Smart Self Management’ intervention	Medication management & symptom recognition	Significant increase in scores for medication management & symptom recognition, p <0.001.		
Benjamin (2017) ‘Smart Self Management’ intervention	Dietary modifications & blood pressure management		Non-significant increase in scores for dietary modifications & blood pressure management, p = 0.2584.	

Only three interventions reported any non-beneficial findings. The My Sister’s Keeper Project and the Way of Faith (WOF) project interventions did not demonstrate a beneficial or protective effect in terms of the isolated outcome of weight reduction [69, 72]. The ‘With Every Heartbeat is Life’ did not show a beneficial change in attitude or intention toward exercise or physical activity [65]. Similarly post-intervention blood sugar increase following intervention in the Way of Faith (WOF) project indicates no beneficial cardiovascular impact. There, however, is a clear demonstration of significantly beneficial impact of the distinct secondary outcomes for hypertension or cardiovascular health, with 14 distinct outcomes showing significantly protective effect and 2 distinct outcomes showing beneficial effect but not to significant levels (Table 7).

### Characteristics of faith institution roles

An outline of the qualitative synthesis based on the Diffusion of Innovation theory, the Communication–Behaviour change model, and the Template for Intervention Description and Replication (TIDieR) Checklist is presented (Table 8).

**Table 8:** Meta-aggregation findings on the features and characteristics of faith institution roles.

**The agents delivering the interventions** The agents delivering hypertension related intervention were, paid or unpaid, trusted based on characteristics as already skilled or trainable, personally influential or health interested, locally-derived and locally-based, personally invested individuals. They were interested adults that qualify as trusted insiders.
**Training received by agents delivering the interventions** The training received by agents consisted interactive, flexible and creative transfer of skills and competencies from experienced role performers, experts or professionals to adult learners of varied abilities. Training was intervention specific, dependent on required roles, theoretically based and pragmatically delivered to enable empowerment of learners with self-development or skill acquisition without formal assessment, enhancement of existing competencies, and sometimes formal, certifiable achievement. Training was customised to the requirements of the intervention and modified as indicated by needs of the adult learners, and as such was characterised by flexibility to accommodate a variety of styles, techniques and abilities.
**Materials used in intervention delivery** The materials used to deliver hypertension interventions comprise communally administered, health content preponderant religion friendly messaging and assets. These include varying combinations of textual and pictorial health and faith content, audio-visual technology assisted health and faith content, and participation-based core religious content delivered with techniques and formats requiring skilled human contact. Locally derived facilitators, influencers and trusted leaders from the faith institutions themselves function as resources and materials for hypertension intervention.
**Component and combinations of the interventions** A broad range of structured group and individual activities targeting the initiation, achievement and maintenance of behaviour change through contextually applicable tools were used. These include: teaching and learning on and linking nutrition, physical activity, spirituality, lifestyle, health and disease; teaching, learning and practice on personal effectiveness and behaviour change; teaching, learning and practice on monitoring cardiovascular health, disease, lifestyle and behaviour; and contextually applicable creative group and cultural activities suited to reinforcement of learning and an active lifestyle, such as music performance and outdoor recreational pursuits.
**Frequency of intervention delivery** The frequency and timing of interventions vary depending on their types, formats and intensities of the intervention activities, resource requirements including training needs, and the local contexts relating to intervention participants. Participant contexts related to urban or rural location, family commitment and employment-determined availability are particularly influential. Intervention durations vary, from six weeks to multiple months, more than a year or several years. Intervention frequencies are as varied as daily or weekly to monthly.
**Religious components of the interventions** The religious components of interventions vary, and faith institutions do contribute roles outside faith or spiritually based activities: such as programme endorsement and publicity, participant recruitment, training and facilitation. Many interventions have no inherent religious component, their host faith institutions being relevant solely in utility as physical assets enabling intervention delivery. Proximity of interventions to routine religious activity does not constitute religious component. The religious components however include: teaching, learning and discussion of religious scripture; incorporation of scripture linking lifestyle, physical and spiritual health; use of prayers, meditation and scriptural reasoning; spiritually based singing, chanting and worship; and the incorporation of unique cultural expressions into spiritual or religious songs.
**Mechanism of action of the interventions** The processes involved in hypertension interventions securing individual and collective behaviour change appear to be through several interrelated and overlapping mechanisms that all involve empowerment and initiation or sustenance of cardiovascular health beneficial actions. The mechanisms are not always explicitly stated, tend to be combined in interventions and include: education, coaching, counselling, reasoning, goal setting, encouragement, motivation, and habit and ritual development.
**Socioeconomic and cultural Compatibility** The interventions had certain features that rendered them trustable and compatible with the prevailing social, economic and cultural values of their target communities. Confidence in some level of pre-existing relationship with key community gatekeepers and the deployment of the Community-based participatory research approach, appear to be constant features. The specific features include the following: ◾ Designs that recognize and respect the spirituality and spiritual values of the target communities, and also incorporate them into the proposed interventions. ◾ Involvement of the target communities in design decisions, promotion of their participation in its delivery and encouragement of a sense of ownership of the intervention. ◾ In pursuing engagement with the target faith communities to secure their participation, according them respect and recognition by working in consultation and collaboration with their established hierarchy. ◾ As much as feasible and achievable in individual contexts, inclusion and appropriate utilisation of local faith institution derived or affiliated experts, decision makers, facilitators and intervention agents. ◾ Deliberate use of culturally appropriate approaches in design and implementation that yield to adaptations informed by the social, economic and environmental realities of the target faith institution users and their communities; and offer participants choice and flexibility. ◾ Use or incorporation of validated tools such as government or professionally validated materials or methods, and trusted secular and religious experts such as healthcare professionals, theologically trained clergy and locally experienced insiders. ◾ Where contextually appropriate, involvement of faith institution and wider community female stakeholders such as pastor’s wives, female clergy, influential gatekeepers and female researchers.
**Clarity of intended benefit** The intended benefits to participants were varied. Their clarity was promoted in several aspects including: promotion and understanding of cardiovascular health issues; provision of opportunity for peer collaboration and professional support in personal cardiovascular risk reduction; empowerment with awareness, knowledge, acquisition of health-beneficial skills; generating motivation for behaviour change; and achievement of beneficial blood pressure, body weight and healthy dietary change outcomes.
**Simplicity of the interventions** The interventions were designed to incorporate simplicity, flexibility and elimination of participant difficulty, whilst focusing on individual participants, encouraging communal implementation and inspiring trust. Included were the sense of communal ownership, empowerment of individual participants, enhancement of active participation, and improvement of accessibility. The feature of sense of communal ownership derive from incorporation of traditional and familiar elements of local culture; use of trusted, influential facilitators and agents for intervention delivery, follow up and encouragement; and customisation to local norms as well as alignment with existing practices including prayers, scripture and faith-based discussion. Individual participant empowerment feature of the interventions derive from the use of transparent and ethical consent processes; the promise and strict maintenance of data confidentiality including by non-inclusion of any data perceivable as sensitive; and where applicable availability of the option for individual participants to extend their participation beyond any basic activities the interventions offer. The features of active participation in the interventions include, in addition to use of familiar or traditional elements from the local culture, generous adaptability of the timing, duration and location of learning or practical sessions; free provision of convenient resources or materials to suit individuals and groups with different abilities and work-life balance; and where applicable, simplification of processes to enable self-administration. The features improving accessibility include the simplification of intervention materials to ensure easy readability and comprehension; and the provision of materials in multiple appropriate languages or dialects.
**Reversibility, perceived risk or trialability of the interventions** The interventions had in-built features that encourage participation by signifying negligible risk to participants, and the reversibility of impacts where participants decide to withdraw prematurely. These features rely on participants’ familiarity with intervention elements, availability of alternative intervention elements, provision of individualised intervention elements, confidence of the clergy in the interventions, and availability of volitional exit. The familiarity features are the intervention elements and activities with which participants were already conversant, and which are perceived as admissible or of negligible risk. The alternative activities features are activities that could replace elements of the intervention where required to secure the participation of particular groups or individuals. The individualised activities features are the activities modified from the intervention elements, to secure participation and engagement of particular individuals who would otherwise not be able to participate. The Clergyman’s confidence feature is the credence invested by faith institution leaders in the intervention- the expression and publicity of which encourages participation. The feature of open exit is the availability to individual participants and groups, of the option of unhindered and volitional self-removal from the intervention without adverse consequences.
**Observability of intervention results** Most interventions did not have features that enabled potential participants to pre-empt, examine or observe potential effects of intervention on earlier participants. When they did, the observability of results by potential faith institution participants were based on open discussion and peer feedback. This involved early intervention participants sharing experiences and new learning with potential future participants, and demonstrating any new attitudes and resulting behaviour.
**Perceived source** Interventions were constructed such that their core messages would be perceived to come from easily identifiable, well-informed, credible sources. The messages of hypertension interventions thus delivered were perceived to derive from four primary sources: healthcare professionals and allied workers; health and healthcare researchers; the teaching from and authority of religious scripture; and, well-informed faith and religious leaders.
**The message** Hypertension interventions were constructed to target the reduction of individual and collective cardiovascular risk, but also include content based on religious traditions and scripture. The core messages of the interventions were clear: cardiovascular risk reduction is achievable by improving health literacy and lifestyle management focussing on nutrition, exercise, stress, sleep and attitudes toward health; and religious scriptures are instructive that divine plans and intentions do exist for sound physical health and healthy living, and to achieve such individuals are responsible and capable. The delivery of the constructed health messages were in sessions that typically lasted 45 to 90 minutes, and were varied in type: from health promotional teaching and scripture infused health topic workshops, to personal individual reflective application guided by learning from teaching and workshops.
**The channel** The channel of delivering cardiovascular health messages included a diversity of media and techniques. These were determined by the contexts of the individual faith institutions and their participants. Media used consist reading materials like handouts, pamphlets, posters, workbooks, manuals, devotionals; digital media including presentations, animations, videos; and interactive media such as church services, prayers, and existing close personal interactions. The techniques varied from class focussed interactive group teaching, discussion and practical demonstrations to individually focussed coaching, motivation, personal reflection and devotion.
**Receiver** Rural and urban resident adults across all income groups were target audiences and eventual receivers of hypertension interventions delivered within faith institutions. Relative to White American, White European, Latino, Haitian American and other minority ethnic backgrounds however, there was a preponderance of Black African, African American eventual targets and receivers of faith institution delivered hypertension interventions.
**Intended destination / outcome** Where explicitly stated the intended outcomes of hypertension interventions included improvement of health literacy and adoption of health promoting behaviour with the outcomes of reduction of body weight and blood pressure.
**Intervention modification** There was no evidence that hypertension interventions delivered within faith institutions underwent modification during the course of the intervention.

Faith institutions roles in hypertension have particular characteristics that hinge on relationships, trust, a sense of ownership, local leadership, a high degree of sociocultural contextualisation and conformity to the practice of faith. Where possible faith institutions used trusted insiders equipped with varied, flexible, faith-friendly materials and direct interpersonal human contact; investing the best available quality context specific training in them. Interventions were flexibly delivered within participants’ contexts, targeted the individual and groups, and comprised or linked lifestyle and spirituality while incorporating or at least respecting their sociocultural and religious realities.

By design the interventions were simplified, transparent, practical and empowering to increase knowledge, give guidance or motivate toward cardiovascular health beneficial habits. The interventions were rationalised as harmless by participants, who then engaged by free choice having perceived the interventions to be respectful, and supported by their faith and community leader.

### Peers and faith related factors are the real drivers of engagement

Sternberg et al. and Lee et al. indicate that the main mechanism of achieving cardiovascular beneficial behaviour change hinges on leveraging the influence of peers and communities within the faith environment to engage in interventions [55, 73].

Sternberg et al. showed that faith-based interventions appear to be influential in reducing cardiovascular risk factors, especially for minorities and women [55]. They found that in order to achieve the outcomes needed to deliver better blood pressure or cardiovascular status, the real drivers of behavior modification are the faith related factors that faith institutions contribute. Among these are pastoral leadership, targeted education and training delivered by members of the faith community. Although the contribution by secular professionals, academia, and governmental and non-governmental agencies in partnership with faith institutions are vital, the required behaviour changes are directly determined by the faith related factors. Similarly, Lee et al. find such faith related factors [73] and peer group influence pivotal in delivering behaviour change that in turn deliver the cardiovascular health benefits. The power of the faith institution peers is influential in engagement with resources, acquisition and fine-tuning of skills needed for personal effectiveness, and the psychological benefit of doing these in the context of a supportive community.

### Faith institution based cardiovascular interventions may be promising

Four studies reported process indicators [56, 62–64]. Baig et al. reflected the view of participants on the motivation for behaviour change delivered by trained lay church leaders to assist them with cardiovascular self-management. They reported high satisfaction rates, with 95% of participants reporting that it was important for them to start classes with prayers. Eighty per cent reported that they had learnt a lot about eating a healthy diet. Seventy per cent reported they had learnt a lot from other participants and felt supported. At the end of the intervention programme 70% felt a lot more confident in discussing their health with medical professionals [62]. Similarly, Draper et al. reported acceptability of the intervention among church pastors, the leaders accepting research partnership with professional researchers on behalf of their congregations, was 100%. Participant satisfaction also received good ratings. Each of the 19 components of the intervention received a “very useful” rating of between 81.4% and 93.0%. The lowest rating of 81.4% was for regular measurement of body weight, and the highest rating of 93.0% was for physical exercise with fellow participants [56].

In Bittman et al. intervention group participants had a significantly higher retention rate of 83.3% compared to control group retention rate of 54.3%, and cardiovascular programme completion was 4.21 times higher for the intervention group compared to control group [63]. Dodani et al. reported a good intervention uptake with 91% of participants attending 7 to 9 sessions out of a 12 sessions and 68% attending 10 to 12 sessions [64].

### Faith institutions are complex, flexible environments for cardiovascular health coaching, motivation and behaviour change

Final synthesis of the qualitative findings on the characteristics of faith institution roles, and the quantitative evidence of the effectiveness of those roles is outlined in Table 9. Participation in the social environment of faith institutions and religious rituals appear to be a form of indirect intervention. The most frequent mechanism of direct cardiovascular intervention was Healthy Lifestyle Coaching, Counselling & Motivation Training, used twice as frequently as the combination of Nutrition and Exercise / Physical Activity, and six times as frequently as nutrition intervention alone. The combination of Exercise / Physical Activity with Healthy Lifestyle Coaching, Counselling & Motivation Training was the least frequently used. Faith institutions are complex assets capable of delivering flexible social environments suited to cardiovascular health coaching / motivation and healthy behaviour change. They are amenable to an eclectic combination of intervention techniques and beneficial hypertension linked outcomes.

**Table 9:** Final synthesis of quantitative indications of effectiveness with the qualitative findings of intervention components and main features.

Categories of interventions by component activities / mechanisms (Number of studies)	Intervention agents	Training received by interventions agents	Intervention target	Channel or technique of delivery	Funding	Frequency of intervention activities	Religious component	Indication of intervention effect
Healthy Lifestyle Coaching, Counselling & Motivation Training 12 studies: [34, 56, 59, 61–63, 65–67, 70, 73, 74]	(1) Trusted researchers, health or healthcare professionals, who deliver the intervention and train others [34, 61, 63, 65, 66] (2) Volunteering faith institution leaders and members who were health or healthcare professionals, and received intervention specific training [61, 63, 73] (3) Volunteering faith institution leaders and members who were not health or healthcare professionals, but received intervention specific training [34, 56, 61, 62, 70, 73] (4) Self delivered intervention activities. [59, 67, 74]	Fully trained and registered professionals received intervention specific, religion friendly training. Training received was intervention-specific, and based on learning theories including Adult Learning Principles, Behavioural Counseling, Motivational Interviewing, Social Cognitive Theory, the Trans-theoretical Model and Self-Determination Theory.	Six were executed in urban areas [34, 61–63, 70, 73]; four in both urban and rural areas [56, 59, 66, 67]; and one in a rural area [65]. Eight programmes targeted all income levels [34, 59, 61–63, 66, 70, 74] and one study targeted low income [56]. Most programmes were directed at mono-ethnic, mono-cultural demographics. Eight targeted Black or African Americans, one targeted Haitian Black Americans, one targeted American Latinos, one targeted Black Africans and one targeted a mixed ethnicity demographic consisting White and Black Americans.	Self-administered reflection or motivation sessions, using devotionals, videos, presentations, animations and reading materials [67, 74]. Small group interactive, direct teaching using tools including presentations, videos, manuals reinforced with post session reading materials [59, 61, 63, 66, 70]. Classroom teaching, practicals and demonstration sessions [34, 56, 62, 73].	Seven programmes were government funded [34, 56, 61, 62, 65, 66, 73], four were funded by non-governmental or non-profit organisations [59, 63, 67, 70], and no source of funding was identified for one programme [74].	Frequency of intervention activities varied by programme. Personal daily intervention activities and weekly individual or small group activities were interspersed with repeated teaching and reinforcements. Programme activities lasted 3 to 4 months and occasionally up to a year.	Clergy endorsed the programmes, supported recruitment drive with public announcements, donated faith institution premises and faith-based social environment. Religious components of two programmes were limited to these [61, 74]. Seven programmes incorporated inspiration from religious scripture in healthy lifestyle lessons, individual meditation, corporate and individual reading, and in music [34, 56, 63, 66, 67, 70, 73]. Seven used prayers [34, 56, 59, 63, 67, 70, 73]. Three incorporated spiritual, gospel or religious scripture based songs [56, 63, 66].	Pooled mean SBP 0.20 mmHg (95%CI -0.44 to +0.49) reduction & DBP 0.04 mmHg (95%CI -0.24 to +0.31) increase at 3 months [34, 62, 63, 68]. Pooled mean BP 0.65 mmHg (95%CI -0.91 to -0.39) reduction & DBP 0.53 mmHg (95%CI -1.86 to 0.80) reduction at 12 months [63, 71]. A trend of significant SBP [56, 61, 74] and DBP [61, 74] reduction; significant DBP increase in one programme [56]; and a mixed picture of SBP and DBP reduction with Whites achieving more significant reductions than Blacks [70]. Pooled mean body weight reduction of 0.12kg (95% CI -0.28 to 0.03) [63, 68, 71] and a polled mean waist circumference reduction of 1.69cm (95% CI -1.74 to -1.64) [62, 63, 71]. Significant knowledge increase on the basics of Hypertension Scores p < 0.001 [59]. Significant increase (*p* < .01) in cardiovascular health knowledge scores [65]. Significant increase in awareness of hypertension and cardiovascular disease as leading cause of death, p = <0.001 [66]. Significant increase in Perceived Personal Control scores, p < 0.0005 [67].
Combination of Nutrition & Exercise / Physical Activity 6 studies [64, 69, 71, 72, 75, 76]	Skilled or trainable insiders, and trusted professionals: (1) Research professionals delivering intervention specific training. (Yanek) (Kinard) (Taylor, 2011) (Dodani, 2014, Dodani, 2013, Dodani, 2015) (2) Qualified health, healthcare and allied health professionals volunteering to receive intervention specific training. (Taylor, 2011) (3) Theologically trained pastors and ministers, and faith institution members volunteering to train as health coaches and advisors. (Yanek) (Kinard) (Taylor, 2011) (Dodani, 2014, Dodani, 2013, Dodani, 2015)	Qualified, registered and practising professionals required were trained in intervention specific procedures (Taylor, 2011). All agents received intervention specific training from the research team (Dodani, 2014, Dodani, 2013, Dodani, 2015), training to deliver nutrition and fitness health promotion curriculum (Yanek, 2001, Kinard, 2016), and blood pressure measurement training delivered by qualified healthcare professionals (Kinard, 2016).	All 6 programmes targeted urban adults of all income groups. One programme targeted all ethnic backgrounds (Taylor, 2011) while the rest targeted Blacks or African Americans (Yanek, Kinard, Dodani, 2014, Dodani, 2013, Dodani, 2015).	Interactive teaching sessions enhanced with visual and practical demonstrations. Post teaching workbooks and handouts.	One programme was funded by a non-profit organisation (Taylor, 2011). Two were government funded (Yanek) (Kinard), and three were funded by a publicly funded university.	Intervention activity sessions were delivered on a weekly basis, and lasted from 7 weeks (Taylor, 2011) or 10 weeks (Kinard), to 12 weeks (Dodani, 2014, Dodani, 2013, Dodani, 2015) and 20 weeks (Yanek). One programme offered an option continuation for up to 36 weeks (Yanek).	Group prayers (Yanek, Kinard, Dodani, Dodani, Dodani). Religious scripture based healthy lifestyle discussion. Religious scripture based music (Kinard).	Significant reduction in SBP & DBP [64, 75, 76]; non-significant reduction in SBP & DBP [72] and non-significant DBP increase [69]. Intervention group mean daily Sodium intake reduction of 145mg/day in daily compared to Control group mean daily Sodium intake reduction of 8 mg/day, p = 0.0167 [71]. Intervention group mean weight decrease of 0.5kg. Control group mean weight increase of 0.38Kg(p = 0.0008) [71]. Intervention group mean SBP decrease of 1.6 mmHg compared to control group mean SBP decrease of 0.95, p = 0.47; Intervention group mean DBP decrease of 0.36 mmHg compared to control group mean DBP increase of 0.22, p = 0.49 [71].
[Indirect intervention] The Social faith environment, Religiosity and Religious Rituals 3 studies [55, 57, 58]	No direct intervention agents (Sternberg) (Sørensen). Qualified medical professionals (Liu)	Not applicable (Sternberg) (Sørensen). Medical professionals trained to measure blood pressure (Liu)	Receivers of indirect intervention included social and family groups from multiple countries (Sternberg), Tibetan Buddhist Monks undergoing hypertension screening (Liu), and Norwegian church attenders (Sørensen).	No direct intervention delivery	Government agency funding (Sørensen) (Liu) and funding by a combination of government bodies and secular bodies (Sternberg).	Routine and regular exposure to religious environments	Social interactions of groups and families within the faith or religious environment (Sternberg) Passive religious routines and the religious environment (Liu) Religious attendance and the social environment within churches (Sørensen)	Behavior modification relies on faith related factors including the spiritual and socio-cultural awareness of agents; making members of the faith community potentially effective agents [55]. Long hours of communal religious rituals and teaching in Tibetan Buddhist settings are associated with a decrease in odds for hypertension [57]. Mean SBP & DBP decreased with increasing Religious Attendance [58].
Nutrition alone 2 studies [60, 77]	Agents delivering the intervention activities included volunteering faith institution leaders and members who were health or healthcare professionals, and received intervention specific training (Wiist)(Ray)	Fully trained dietician / nutrition professionals and practicing nurses (Ray). Religion compatible nutrition training was given to non-professional volunteers (Wiist).	Both programmes were directed at African Americans, executed in rural and urban areas, and targeted all income levels (Wiist, Ray).	Teaching sessions aided with posters, manuals and post teaching pamphlets and other reading materials (Ray, Wiist)	Wiist was state funded. Funding for ray was not disclosed.	Intervention frequencies were design specific, with intensive contact over 3 weeks of 16-week programme (Ray) and six weekly intervention contacts (Wiist).	Clergy endorsed the programmes. Faith institution assets were used as classrooms (Wiist), and religious scripture was utilised in healthy nutrition lessons (Ray).	Significant reduction in the weight of intervention participants, F(3,12) = 5.29, p<0.05 compared to non-significant weight increase in non-participants. Significant reduction in SBP & DBP [77]. Significant cholesterol levels decrease for participants (233.9 to 210.4mg/dl, p<0.0001), similar to cholesterol levels decrease for Controls (241.5 to 202.9mg/dl, p<0.0001) [60].
Combination of Exercise / Physical Activity with Healthy Lifestyle Coaching, Counselling & Motivation Training 1 study [68]	Agents delivering the intervention activities included volunteering faith institution leaders and members who were health or healthcare professionals (Tucker)	Training received by intervention agents included project specific coaching and data collection training (Tucker).	The programme was directed at urban African Americans of all income levels (Tucker).	Delivery of learning was via individual coaching and physical activity sessions; and group and panel discussions (Tucker).	Non-profit organisation	Flexible individualised coaching, 6 weekly physical activity sessions, 6 weekly group discussions	Recruitment of intervention agents, endorsement of programme, and recruitment of participants.	Non-significant SBP reduction (2.91mmHg, F = 2.48, p = p = 0.117 and Control group SBP reduction of 1.83 mmHg, F = 1.06, p = 0.303). Non-significant DBP reduction (0.3mmHg -no change-, F = 0.06, p = 0.815 and Control group 2.17 mmHg decrease, F = 3.18, p = 0.076). Non-significant weight decrease (1.69 Lbs., F = 2.95, p = 0.087, and Control group non-significant decrease of 0.97 Lbs., F = 1.07, p = 0.303). Significant increase in Nutrition label literacy (1.2 units, F = 30.89, p<0.001 v Control group non-significant decrease of 0.06 units, F = 0.09, p = 0.76). Significant increase in Healthy Eating score (0.28, F = 26.32, p<0.001 v control group score increase of 0.08, F = 2.69, p = 0.103). Increased Healthy Drinking score (0.88, F = 18.75, p<0.001 v control group score increase of 0.21, F = 1.40, p = 0.239). Increased Physical Activity score (0.30, F = 20.87, p<0.001 v control group Physical Activity score of 0.19, F = 10.95, p<0.01.) Increased overall level of engagement in health- smart behaviours (increase of 0.76, F = 26.47, p < .001 v Control group increase of 0.30, F = 5.33, p = 0.022).
Combination of Nutrition & Exercise / Physical Activity & Lifestyle health coaching, counselling, motivation training (No studies)								
Exercise / Physical Activity alone (No studies)								

### Quality of the evidence

All the studies contributed to the qualitative synthesis. Two studies were adjudged with serious or high overall risk of bias, however the characteristics of the interventions synthesised are exclusive to the subdomains contributing to those bias ratings. Thus, the qualitative findings, can be held with confidence, as they did not contribute to any adjudged serious / high risk of bias.

## Discussion

To facilitate achievement or maintenance of normal blood pressures, faith institutions purposefully assume roles in cardiovascular health promotion and blood pressure measurement. Deployed in a variety of innovative ways these comprise: cardiovascular health and disease knowledge teaching, with illustrative linking to lifestyle; facilitation of exercise or physical activity as part of normal lifestyle; facilitation of diet and nutrition change beneficial for cardiovascular health; and cardiovascular health linked measurements. In addition faith institution roles include encouragement of personal psychological control, and opportunistic blood pressure checks. Within intervention programmes, these roles tend to be deployed in combination rather than in isolation. Healthy lifestyle coaching, counselling and motivation training was a more frequently employed mechanism compared to physical activity and nutrition or dietary change.

Globally faith institutions have formed partnerships with healthcare systems and contributed to addressing general unmet general healthcare and chronic disease preventative needs of communities [78–83]. Findings of this review are in agreement with the literature on the well-established record of faith institutions contributing to the implementation of public health interventions, including those attempting to influence behaviour change [84–87].

This review sheds light on faith institution roles, identifying the particular characteristics that those roles must posses: characteristics contributory to why and probably how the mechanisms of behaviour change of interventions work. They include relationships of trust, a collective sense of ownership, an informed leadership derived from the faith institution and the local community, sociocultural contextualisation of the roles or their constituent activities, and conformity of such to the practice of faith.

Of particular relevance is the constant pivotal phenomenon of leaders and influencers within these institutions being probably the most determinant resource [88–90]. The literature is largely focused on cultural and spiritual considerations, intervention design, community engagement, implementation strategies for successful outcomes and contributing recommendations [87, 91, 92]. Peterson et al. however, identified the key elements church-based health promotion programmes require to facilitate successful outcomes, albeit without in-depth discussion or investigation of the characteristics of those elements [93]. They include formation of partnerships between faith and health organisations, positive values held by faith organisations and their leaders, availability within faith organisations of adopted or existing health-related services, access of churches to members able to be engaged in health promoting roles, community focus of churches, inherent capacity to promote health behaviour change, and inherent supportive relationships. The identified elements and characteristics of church-based interventions indicate desirable conditions and characteristics for wider faith-based and faith-placed interventions. They overlap in scope with and are in concordance with the findings of this review. There however were until this review, no identified systematic reviews on the characteristics of those roles or elements of interventions generally, or specifically directed to mitigate hypertension within faith institutions.

This review demonstrates agreement with the general trend of transparency and deliberate, purposeful stakeholder engagement through the approach of Community-based participatory research [94]. This approach involves attention to cultural sensitivity, evidenced by a particularly strong representation of faith communities in formulating interventions, training of volunteers, engagement of contractors, and intervention delivery. Using this approach, researchers, academics and clinical practitioners engaged the religious and faith institutions as partners and full participants. For example, in delivering general health education or specific preventative interventions, research practice has evolved to utilise the internal social networks of people of faith and casual attenders at faith institutions [87, 95]. The use of this approach in studies constituting this review is in keeping with evidence from research practice involving interventions for conditions as diverse as hypertension, diabetes, cancers, and HIV-AIDS [88, 96–98]. With respect to the characteristics of roles played by faith institutions, no areas of disagreement were identified between the findings of this review and existing literature. This may be related to the relative limited literature in the area.

The evidence of effectiveness of faith institution roles is evolving. In the randomised controlled studies, after three months of intervention, there was the small benefit of a non-significant systolic blood pressure reduction, albeit with a non-significant increase in diastolic blood pressure. Over the longer duration of 12 months however, there is persistent and significant systolic blood pressure reduction and a non-significant diastolic blood pressure reduction. There were only a few randomised controlled studies contributing to the meta-analyses, indicating that further research is needed. Nonrandomised studies findings are in agreement with the randomised studies, indicating that the interventions have the generally beneficial and significant effect of lowering systolic and diastolic blood pressures. Similarly for body weight and waist circumference, meta-analyses show an overall beneficial albeit non-significant reduction. Non-aggregated evidence from the non-randomised studies show that distinct biological outcomes targeted by the interventions had individually significantly beneficial effects on cardiovascular health and hypertension. That is, they are protective or beneficial for cardiovascular health and hypertension. These findings are in keeping with the well-established record of faith institutions in contributing to implementation of public health interventions. Spirituality and religious participation–both typically facilitated by faith institutions—are known to be capable of both beneficial and harmful influences on health [99]. And there is significant literature support for the favourable effect of religious participation on personal wellbeing [100–102]. For chronic diseases including cardiovascular problems, more favourable outcomes are associated with spirituality [103], faith-based nature of interventions [104] and direct involvement of faith institution in delivery of interventions [25, 105]. For example there is evidence that short-term effectiveness of health behaviour interventions implemented utilising ordinary or typical users of faith institutions, notably church members as volunteer counsellors, advisers and interventionists, lead to increased physical activity and better dietary change outcomes [106–108]. Although there remain knowledge gaps with regard to individual chronic diseases, there is evidence that the environment within faith-organisations is generally favourable to health promotion [109]; and cardiovascular related and obesity interventions implemented within faith-based organisations have been successful in achieving weight reduction, dietary improvement and increased physical activity [110].

The process indicator outcomes in this review may be indicative of potential future reception of interventions facilitated within the faith environment. Where available, participant retention, intervention uptake, acceptability and satisfaction rates were positive. These are probably reflective of the community based participatory approach adopted in constructing the interventions. This approach was a feature common to the studies contributing to this review. Rather than engagement as research specimen, local communities through faith institutions were decision, research, and implementation partners. This aligns with the literature that full community participation is not only beneficial to enhance the quality of research through direct stakeholder facilitation, but also for the relevance and sustainability of any implementation [111–113].

The evidence contributing to this review derived from a plurality of settings where the participants were Black African and African American Christian adults. This is perhaps unsurprising, keeping with longstanding greater frequency and worse outcomes of hypertension in Blacks compared to non-black peoples in the USA [114]; a profile that fits hypertension in African people outside the USA [115]. Especially since African American religious settings and leaders have used their influence to address socio-political and health disparities [23, 116], and widely engaged in research and healthcare partnerships to benefit their communities [117–120].

The interventions did not provide for continuation of blood pressure screening or on-going measurements following the end of the programmes of intervention. Similarly, although the benefits of the health promotion were intended to be lasting, there was no evidence of provision for routine or on-going personal measurement of blood pressure beyond the end of the programmes of intervention. Also, despite the blood pressure measurements being used as opportunity to initiate cardiovascular health beneficial behaviour change, there appeared to be lack of emphasis on the importance of routine and on-going blood pressure measurements as part of normal lifestyle. These may be partly due to long term implementation challenges, especially since resource constraints are a key hindrance to the long-term sustainability of community-based cardiovascular interventions including low cost and effective ones [121]. And, sustainability is generally complex and intervention dependent [122].

Access to healthcare systems for research participants was generally not addressed, and there were no attempts to link up or incorporate faith institution health promotion with the wider healthcare systems. This is a probable indication of the relative ease of access to healthcare in the well-resourced countries where most of the studies were conducted. With respect to faith institution facilitated cardiovascular or hypertension intervention, access to health was not addressed as an intrinsic healthcare system issue. This reflects the fact that nongovernmental, faith or philanthropic healthcare is most likely to be part of the structure in low income countries or those with fragile health systems [19, 123]. Similarly there was no emphasis on the integration of faith institution functions with the health system, a reflection of the resource levels and non-reliance on institutions outside the health system, such as faith institutions.

Ultimately, the size or orthodoxy of invested resources may not be the most impactful determinants of the magnitude of cardiovascular health gains achievable within or through the faith environment. But, probably, in the presence of trust, the gains may be ultimately conditional upon processes incorporating deliberate cultural awareness, immersive involvement of the leaders, respect for the local religious context, and compatibility with the continuation of religious worship. These can be tough to achieve without inside knowledge of the explicit characteristics of individual intervention activities that faith institutions, as unique entities, can agree to on behalf of their patrons and the larger society.

### Limitations

There is inherent language bias, as the search was conducted in and all the publications were in English. Similarly, publication bias cannot be ruled out. It is a given that successful programmes and interventions are more likely to achieve publication. All the programmes and interventions reported an overall positive impact, although to varying degrees. Not a single publication reported an overwhelmingly ineffective intervention or programme.

Otherwise, weaknesses indicated in the findings of this review are in five specific areas.

One: There is a paucity of cardiovascular research literature specifically dealing with faith institution facilitated hypertension interventions. Two: The absence of studies from Low to middle income countries including the Africa region, Asia and Latin America is an important limitation. This is made more important by the fact that religious affiliation and attendance continue to be an important component of social life in those regions. Three: The absence of hypertension intervention studies from Islamic institutions or targeting Muslim congregations constitutes an important limitation. Islam is a major world religion practiced by significant proportion of populations in every WHO region. Islamic inspired established community serving primary and general healthcare programmes do exist, however absence of any that satisfy the inclusion criteria of this review is perhaps indicative of the current dearth of hypertension intervention literature deployed within faith institutions, an underrepresentation of such in the context of the Islamic faith, and perhaps other factors yet to be identified. For example, the authors of this review were unable to successfully obtain hypertension specific programme information from one of such—the Chicago and Atlanta based Inner-City Muslim Action Network (IMAN) [124].

Four: An overwhelming majority of the studies are from the USA, imposing a limitation on sub-group analyses by countries and WHO regions. Five: The predominant context addressed in the included studies is that of Christianity and Christianity affiliated or professing African Americans, who do have some access to various health systems within the USA. This context is not universally applicable.

### Strengths of the review

To the best of our knowledge, this is the first systematic review to focus on the roles played by faith institutions on hypertension and cardiovascular health of adults, and the characteristics of those roles.

A particular strength of the review lies in the use of the diffusion of innovation framework, a naturally occurring social knowledge transfer process nearly indistinguishable from how faith institutions have naturally operated; and hence a most natural fit for the context. The findings of this review indicate generalisability to contexts of religious participation within urban and rural settings involving adults across all income levels.

In addition there are four unique areas of strength demonstrated by the findings of this review. First: Due to the provenance of the included studies, the findings are potentially compatible with: contexts of state funding or charitable organisation funding of hypertension or cardiovascular research; contexts of well organised and funded health research infrastructure; and contexts where healthcare systems prioritise hypertension or cardiovascular research. Second: The findings are potentially particularly helpful in contexts where religious participation is cultural, socially valued or popular. In these contexts faith institutions could potentially be utilised as viable assets or adjuncts for healthcare systems. Third: The findings potentially have strong applicability to settings bearing similarities to the African American socioeconomic and cultural context, including nutritional, cultural and religious legacies. Four: The findings of the review are potentially particularly useful in contexts where barriers to good cardiovascular health are preponderant. These include populations with high rates or religiosity, urban habitation, inadequate healthcare system access, inadequate hypertension awareness, poor nutrition, poor physical activity, cultural insularity or isolation, poverty or low income and remote, rural communities.

### Implications for research

The findings and observations from this review highlight the need for further research in three areas.

One area of research need is exploration of how to systematize, routinize and normalize blood pressure measurement within faith institutions such that users of faith institutions have constant and permanent access to blood pressure measurements outside of and unrelated to any on-going research activity. Another area of research need is exploration of how healthcare systems at different resource levels can utilise the learning from faith institution based cardiovascular health promotion for early diagnosis, management and other relevant intervention. Finally, research is needed on understanding the barriers, facilitators and feasibility of integration of faith institution health promotion into wider healthcare systems. Example of such could be hypertension referral systems, or on-going support for chronic disease or cardiovascular disease preventative activity within faith institutions.

### Implications for cardiovascular intervention within faith communities

This review agrees with current knowledge on hypertension and cardiovascular interventions within faith institutions that: the faith institution environment is potent resource, religiosity and the practice of faith contribute to the achievement of intervention outcomes, and religious leaders are probably the most influential facilitating factor.

This review contributes the following update to the literature. There is a broad range of roles but they probably are most impactful when their characteristics are purposefully adjusted and highly contextualised to the individual faith setting. These done, small effects beneficial to hypertension and cardiovascular health will probably accrue over time. For maximal impact hypertension and cardiovascular interventions have the following requirements: contextualisation to local circumstances, lifestyle linked hypertension and cardiovascular knowledge impartation, designs incorporating psychological empowerment, designs incorporating multiple measurement of cardiovascular health related parameters, opportunistic hypertension checks in addition to other blood pressure measurements, full involvement of locally extracted professionals, faith and community leaders taking responsibility for all aspects of intervention, and implementation without the disruption of religious practice or on-going religiosity.

The findings of the review also suggest that there is the need for involvement in cardiovascular and hypertension research of more diverse faith and religious cultures, traditions and environments. Similarly, there is the need to understand how faith institutions, in seeking involvement in cardiovascular health and hypertension, compare to and can benefit from non-faith-based institutions.

## Conclusion

Pertinent contribution to knowledge and the key messages for communities globally, policy makers, healthcare professionals, researchers and other stakeholders are highlighted in Table 10.

**Table 10:** Key messages.

**Roles of faith institutions**
**To assist adults achieve or maintain a normal blood pressure, faith institutions play a variety of roles including:** • Cardiovascular health and disease teaching, with direct linking of daily lifestyle to health and disease. • Promotion of, provision for, and persuasion to exercise or increase physical activity as part of normal lifestyle. • Promotion of, provision for, and persuasion toward diet and nutrition change beneficial for cardiovascular health. • Promotion of, provision for, and persuasion to undertake cardiovascular health linked measurements. • Teaching, training and encouragement of personal psychological control. • Promotion of, provision for, persuasion to undergo opportunistic blood pressure checks.
**Characteristics of the roles**
**Faith institutions activities that assist adults achieve or maintain normal blood pressure:** • Are based on relationships of trust with local leadership • Are contributed to by trusted local insiders and leaders • Foster a sense of ownership • Require simplification, transparency, and health-lifestyle-spirituality linking • Are highly contextualised to individual local sociocultural realities • Work alongside and are in conformity with the practice of faith • Involve investment of training in or cooperation with trusted insiders • Are ethical and harmless to participants • Are volitional but consented to by faith and community leaders
**Evidence of Effectiveness**
• The evidence for effectiveness is limited. • Faith institution activities cause reductions in systolic and diastolic blood pressures; but these reductions appear to become less significant over time. • With intervention, body weight and waist circumference reduce. Similarly, multiple, health related outcomes are also impacted in a way beneficial for hypertension and cardiovascular health.
**Other findings**
• The literature is limited. • Interventions work through multiple components; and the quantification or separation of their relative contributions is not straightforward. • Within intervention programmes, faith institution roles tend to be deployed as combination of mechanisms rather than in isolation. • The most frequent mechanism was Healthy Lifestyle Coaching, Counselling & Motivation Training. • Interventions work in all socioeconomic, faith and resource settings; but more work is required to further understanding. • Current evidence is predominantly based on research on Black African and African Americans in the Christian faith. • Where available evidence shows high and encouraging rates of intervention uptake, retention, satisfaction and acceptability. • Community and peer influences appear to be an important mechanism of achieving beneficial behaviour change. • More research is needed across different faith, resource and health system settings.

Faith institutions contribute a variety of roles to assist adults achieve or maintain normal blood pressures. Certain characteristics are important for the feasibility and outcome of hypertension and cardiovascular interventions. Most of the evidence derives from settings with predominantly Black African and African American Christian adults. This implies potentially limited generalizability. However, where applicable, for example in contexts of deprivation of cardiovascular preventative health where religious attendance or participation is prevalent, these findings are invaluable for the prospects of developing low cost, effective and sustainable cardiovascular interventions. Such contexts exist throughout low and middle-income countries globally, and also in pockets of deprivation in high-income countries.

However limited, there is evidence of effectiveness of faith institution facilitated interventions. Faith settings may be amenable to tailored cardiovascular coaching and motivation to specifically address hypertension. Contextualised and tailored innovative application of interventions are attainable, with potentially beneficial outcomes.

Although cultural and religious influences on human behaviour vary across communities globally, this review contributes evidence on faith institution roles and the characteristics of those roles for beneficial cardiovascular public health intervention. These are potentially useful for the construction of community based, long-term, meaningful, sustainable, and perhaps permanent interventions.

In addressing the global hypertension epidemic cardiovascular health promotion roles of faith institutions probably hold unrealised potential in research and utility as viable assets or adjuncts to healthcare systems, crucially in low income, religious or underserved communities across different healthcare settings. Utilising available evidence to construct and deliver bespoke, sustainable interventions that remain effective and stand the test of time remains a challenge.

## Supporting information

S1 Checklist PRISMA 2020 checklist. (DOCX)
